# Chromosome-level genome assembly of Hamlin sweet orange and transcriptomic insights into MADS-box gene regulation of secondary fruit development

**DOI:** 10.3389/fpls.2026.1925055

**Published:** 2026-09-08

**Authors:** Jiaxing Wan, Zhuozhuo Wu, Yanji Yao, Wanshan Zhang, Yuxia Gao

**Affiliations:** 1 National Navel Orange Engineering Research Center, Gannan Normal University, Ganzhou, Jiangxi, China; 2 Jiangxi Provincial Key Laboratory of Pest and Disease Control of Featured Horticultural Plants (2024SSY04181), Ganzhou, Jiangxi, China

**Keywords:** comparative genomics, genome assembly, MADS-box, secondary fruit, sweet orange, WGCNA

## Abstract

**Introduction:**

The MADS-box gene family comprises key transcription factors that regulate plant growth and development, particularly flower and fruit morphogenesis. However, the evolutionary characteristics of this gene family in sweet orange (*Citrus sinensis*) and their specific regulatory roles in secondary fruit development remain unclear. This study aimed to systematically investigate these aspects by comparing cultivars with distinct fruit phenotypes.

**Methods:**

Newhall navel orange (*C. sinensis* ‘Newhall’), which exhibits a typical secondary fruit phenotype, and Hamlin sweet orange (*C. sinensis* ‘Hamlin’), which lacks this trait, were used as experimental materials. A high-quality, chromosome-level *de novo* genome assembly was generated for Hamlin sweet orange. Comparative genomic analysis was performed to identify drivers of divergence between the two cultivars. Additionally, transcriptomic sequencing was conducted across bud, full bloom, and post-bloom stages, followed by weighted gene co-expression network analysis (WGCNA) to identify gene modules associated with secondary fruit development.

**Results:**

The Hamlin sweet orange genome assembly achieved an N50 of 32.89 Mb. Comparative genomics revealed that tandem duplication is a primary driver of divergence between the two cultivars. Gene family identification showed that Newhall possesses more MADS-box genes (139) than Hamlin (119). Transcriptomic and WGCNA analyses identified gene modules significantly correlated with secondary fruit development, pinpointing CSN*SEP*3 and CSN*AG*6 as key candidate regulatory genes.

**Discussion:**

This study provides a high-quality reference genome for Hamlin sweet orange and offers valuable transcriptomic insights into the role of MADS-box genes in citrus differentiation and fruit morphogenesis. The identification of CSN*SEP*3 and CSN*AG*6 as critical regulators of secondary fruit development lays a theoretical foundation for molecular breeding programs targeting fruit morphology in citrus.

## 1 Introduction

During the plant life cycle, the transition from vegetative to reproductive growth is a critical determinant of successful reproduction, with flower development and morphogenesis representing the pinnacle of this process. As a highly conserved family of transcription factors, MADS-box genes play a pivotal role in regulating various stages of plant growth and development, particularly in determining floral organ determinacy and identity (Ning et al., 2023). The family derives its name from its four founding members: MCM1 in budding yeast (*Saccharomyces cerevisiae*), AGAMOUS (*AG*) in thale cress (*Arabidopsis thaliana*), DEFICIENS (*DEF*) in snapdragon (*Antirrhinum majus*), and Serum Response Factor (*SRF*) in humans (*Homo sapiens*) (Ghorbani Marghashi et al., 2020; Ning et al., 2023). These proteins contain a highly conserved MADS domain of approximately 60 amino acids, which functions as a *cis*-acting element by binding to the promoter or enhancer regions of target genes to regulate their transcriptional expression (Smaczniak et al., 2012; Ning et al., 2023).

Beyond their role as floral organ identity genes in the classical ABCDE model, MADS-box genes constitute a complex regulatory network that spans the entire plant life cycle (Ning et al., 2023). During the vegetative stage, they regulate root development (*OsMADS27* in rice), plant architecture, and flowering time (Alfatih et al., 2023; Zhang et al., 2023; Duan et al., 2024). During reproductive development, they finely orchestrate fruit development and ripening; for instance, *AcMADS32* in kiwifruit influences carotenoid accumulation, MADS-box genes in pineapple coordinately regulate fruit post-ripening, and the *MbRIN* gene controls fruit softening in banana (Hu et al., 2021; Lakhwani et al., 2022; Lin et al., 2023). Furthermore, family members serve as crucial hubs in abiotic stress responses. *CsMADS-box* genes in *Camelina sativa* respond to drought stress, *GmMADS3* in soybean enhances salt tolerance, and MADS-box genes in guayule (*Parthenium argentatum*) and rubber tree (*Hevea brasiliensis*) mediate responses to multiple environmental stresses. These findings underscore the central role of this gene family in linking environmental signals with internal developmental regulation (Chen et al., 2023; Tahmasebi et al., 2025; Qu et al., 2026).

The MADS-box gene family exhibits remarkable diversity and complexity in protein sequence and structure, providing the foundation for functional diversification and fine-tuned regulation. Based on protein domain composition and phylogenetic relationships, family members are broadly classified into Type I and Type II (Ning et al., 2023). Type I genes possess a relatively simple structure, typically containing only the MADS domain, and are primarily involved in gametophyte development, endosperm formation, and stress responses (Qiu et al., 2024). Type II genes feature a more complex architecture comprising MADS, K-box, and C-terminal domains, and can be further subdivided into MIKC* and MIKC^C^ subtypes. MIKC^C^-type genes play a central role in reproductive processes such as floral organ development, whereas MIKC*-type genes predominantly regulate male gametophyte development (Ning et al., 2023; Qiu et al., 2024).

Research on the MADS-box gene family has expanded from model plants to various species of economic and evolutionary significance, revealing both universal evolutionary patterns and species-specific characteristics. Comparative studies across diverse plant species have demonstrated significant variations in gene number, type distribution, expansion mechanisms, and functional diversification. The substantial variation in total gene numbers among species reflects the influence of genome size and the frequency of duplication events (Lakhwani et al., 2022; Lin et al., 2023; Ning et al., 2023; Duan et al., 2024). Type distribution exhibits two typical patterns: Type II genes predominate in apple and kiwifruit, whereas Type I genes are predominant in rambutan and *Coptis chinensis*, suggesting that the two major branches of the MADS-box family have undergone divergent evolutionary trajectories across different plant lineages (Lakhwani et al., 2022; Lin et al., 2023; Ning et al., 2023; Duan et al., 2024). Regarding expansion mechanisms, whole-genome duplication (WGD) and tandem duplication (TD) are ubiquitous driving forces, though their relative contributions vary among species. Functional studies have revealed a unity of conservation and novelty: the core regulatory functions in floral organ development are highly conserved across the plant kingdom, with multiple genes in *C. chinensis* being orthologous to *AG*, *SEP*, and *AP3/PI* in *Arabidopsis*. Concurrently, species-specific functions continually emerge; for example, MADS-box genes in rice participate in plant height regulation, root development, and various stress responses; *AcMADS32* in kiwifruit regulates carotenoid accumulation, directly impacting fruit quality; and MIKC^C^-type genes in apple play critical roles in anther development and flowering time regulation (Lakhwani et al., 2022; Lin et al., 2023; Ning et al., 2023; Duan et al., 2024). These comparative studies not only elucidate the evolutionary dynamics of the MADS-box gene family but also provide a crucial theoretical foundation for understanding the molecular basis of plant morphological diversity and for the molecular design breeding of crops.

Citrus is a globally significant economic crop, valued not only for its high nutritional content and unique flavor but also for its positive role in maintaining agricultural ecological diversity (Abeysinghe et al., 2007). Among the numerous citrus cultivars, Newhall navel orange stands out as a premier fresh-eating sweet orange due to its superior quality, rich aroma, and high market acceptance. The secondary fruit is the most distinctive morphological hallmark of navel oranges, from which the name “navel” is derived (Lima and Davies, 1984). Morphologically, the secondary fruit is a small secondary fruit located at the apex of the primary fruit, developing from an additional ovary, and occasionally containing vestigial remnants of the stigma and style (Lima and Davies, 1984; Cerri and Reale, 2020). From the perspective of floral organ development, the formation of the secondary fruit is essentially the result of an increased number of carpels (Bobrov and Romanov, 2019). While normal citrus flowers develop an ovary from a single carpel whorl, navel orange flowers form multiple carpel whorls during differentiation: the primary carpel whorl develops into the main fruit, whereas the secondary carpel whorl develops into the secondary fruit (Bobrov and Romanov, 2019). Notably, the secondary fruit is a trait unique to navel oranges, lacking a corresponding phenotype in model plants such as *Arabidopsis*; this specificity has resulted in a relative scarcity of related research.

In this study, we utilized a pair of phenotypically contrasting cultivars within the Washington navel orange lineage—Newhall navel orange (CSN)(with navel) and Hamlin sweet orange (CSH)(without navel)—as a model system. We aimed to characterize the genome-wide features of the MADS-box gene family in sweet orange and investigate the differential expression patterns across various stages of flower development to identify key candidate genes associated with the secondary fruit trait. Specifically, we first performed the *de novo* assembly and annotation of the diploid genome of Hamlin sweet orange. Subsequently, we conducted a genome-wide identification of MADS-box genes in both CSN and Hamlin sweet orange. Integrating these genomic data with transcriptomic sequencing analysis, we identified two key candidate genes critical for secondary fruit development in Newhall navel orange, thereby providing a theoretical foundation for trait improvement and molecular breeding in citrus.

## 2 Materials and methods

### 2.1 Plant materials

In this study, the genome of Hamlin sweet orange was sequenced. Genomic DNA was extracted from plant materials collected at the National Navel Orange Engineering and Technology Research Center germplasm resource nursery in Ganzhou, Jiangxi Province, China. For the transcriptome analysis, samples of CSN and Hamlin sweet orange were cultivated in Xinfeng County, Ganzhou, Jiangxi Province. To capture the dynamic process of flower and early fruit development, samples were collected at three stages: flower bud stage, full bloom stage, and petal fall stage. For each sampling event, at least five healthy trees with consistent vigor were selected, and flower or young fruit tissues exhibiting uniform developmental stages and free of pests and diseases were collected from different orientations of the tree canopy. For each cultivar and each stage, three independent biological replicates were established, with each replicate comprising a pooled sample from multiple trees. Immediately after collection, samples were flash-frozen in liquid nitrogen and stored at -80 °C for subsequent total RNA and endogenous hormones extraction.

### 2.2 DNA and RNA extraction

Genomic DNA was extracted using the phenol-chloroform method as previously published (Rana et al., 2019). The quality of genomic DNA was evaluated using agarose gel electrophoresis and a Qubit 3.0 Fluorometer (Life Technologies, USA) to ensure the DNA met the requirements for library construction. Fresh roots and leaves were collected separately for RNA extraction. Total RNA from two samples was extracted using the RNAprep pure Plant Kit (TIANGEN, China). Both agarose gel electrophoresis and Nanodrop were used to test the purity and degree of RNA degradation. The RNA quality and quantity were assessed using Qubit and Agilent 2100. The same method was employed for the extraction and purification of RNA from the 18 samples (2 cultivars, 3 stages, 3 replicates) used for the transcriptome analysis of navel orange secondary fruit development.

### 2.3 *De novo* genome assembly

Hifiasm v0.15.4 (Cheng et al., 2021) with default parameters was used to assemble HiFi reads into contigs. Based on Hi-C data obtained from sequencing, the assembled contig sequences were anchored to the near-chromosome level using ALLHIC v0.9.8 (Zhang et al., 2019). The assembly was then manually corrected according to chromosome interaction intensity using Juicebox v1.11.08 (Durand et al., 2016) to produce a chromosome-level genome.

### 2.4 Repeat annotation

A combined strategy based on homology alignment and *de novo* search was applied to identify genome-wide repeats. Tandem Repeats were extracted using TRF (Benson, 1999) for ab initio prediction. For homology-based prediction, the Repbase database (Jurka et al., 2005) was employed using RepeatMasker software (Tempel, 2012) and its in-house scripts (RepeatProteinMask) with default parameters to extract repeat regions. For ab initio prediction, a *de novo* repetitive elements database was constructed using LTR_FINDER (Xu and Wang, 2007), RepeatScout (Lian et al., 2015), and RepeatModeler (Flynn et al., 2020) with default parameters. All repeat sequences longer than 100 bp with gaps (‘N’) accounting for less than 5% constituted the raw transposable element (TE) library. A custom library (a combination of Repbase and our *de novo* TE library, processed by uclust to yield a non-redundant library) was supplied to RepeatMasker for DNA-level repeat identification.

### 2.5 Structure annotation

Structural annotation of the genome was performed using a consensus approach incorporating ab initio prediction, homology-based prediction, and RNA-Seq assisted prediction to annotate gene models. Sequences of homologous proteins were downloaded from the Citrus Genome Database. Protein sequences were aligned to the genome using Tblastn v2.2.26 (Camacho et al., 2009) with a threshold of E-value ≤ 1e−5 (Gertz et al., 2006). The matching proteins were then aligned to the homologous genome sequences for accurate spliced alignments using GeneWise (v2.4.1) (Birney, 2004) to predict gene structures contained in each protein region. For ab initio gene prediction, Augustus v3.2.3 (Nachtweide and Stanke, 2019), Geneid v1.4 (Alioto et al., 2018), Genescan v1.0 (Burge and Karlin, 1997), GlimmerHMM v3.04 (Majoros et al., 2004), and SNAP (Korf, 2004) were used in the automated gene prediction pipeline.

Iso-seq data were processed using SMRT (Lou et al., 2020) link 5.0 software to generate Circular Consensus Sequences (CCS). Additionally, six RNA-seq datasets of fruit tissue for CSN (Qiao et al., 2017) were downloaded from the NCBI database (SRA numbers: SRS1303581, SRS1303583, SRS1303585, SRS1303594, SRS1303595, SRS1303596). Transcriptome read assemblies were generated with Trinity v2.1.1 (Haas et al., 2013; Grabherr et al., 2011) for genome annotation. To optimize the genome annotation, RNA-Seq reads from different tissues were aligned to the genome sequences using Hisat v2.0.4 (Kim et al., 2015) with default parameters to identify exon regions and splice positions. The alignment results were then used as input for Stringtie v1.3.3 (Pertea et al., 2015) with default parameters for genome-based transcript assembly.

A non-redundant reference gene set was generated by merging genes predicted by the three methods using Evidence Modeler (EVM) v1.1.1 (Haas et al., 2008), incorporating Program to Assemble Spliced Alignment (PASA) (Haas, 2003) terminal exon support and including masked transposable elements as input into gene prediction. Individual gene families of interest were selected for further manual curation by relevant experts.

### 2.6 Functional annotations

Gene functions were assigned according to the best match by aligning protein sequences to the Swiss-Prot database (Boutet et al., 2007) using Blastp (Camacho et al., 2009) with a threshold of E-value ≤ 1e−5 (Gertz et al., 2006). Protein motifs and domains were annotated using InterProScan70 v5.31 (Quevillon et al., 2005) by searching against publicly available databases, including ProDom (Servant et al., 2002), PRINTS (Attwood et al., 1994), Pfam (Mistry et al., 2021), SMART (Lei et al., 2022), PANTHER (Mi et al., 2019), and PROSITE (Hulo, 2006). Gene Ontology (GO) (Schlicker et al., 2010) IDs for each gene were assigned according to the corresponding InterPro (Hunter et al., 2009) entry.

Protein functions were also predicted by transferring annotations from the closest BLAST hit with a threshold of E-value < 1e−5 in the Swiss-Prot database (Boutet et al., 2007) and using DIAMOND v0.8.22 (Buchfink et al., 2015) against the NR database (Pruitt et al., 2007). Furthermore, the gene set was mapped to KEGG pathways (Kanehisa et al., 2017) to identify the best match for each gene.

### 2.7 Non-coding RNA annotation

tRNAs were predicted using tRNAscan-SE Online (Lowe and Chan, 2016). For rRNAs, which are highly conserved, prediction was performed using Blastn (Camacho et al., 2009) with rRNA sequences of *Citrus sinensis* Valencia as references. Non-coding RNA genes were annotated using the Rfam 14.0 database (Kalvari et al., 2020), including miRNAs and snRNAs, were identified by searching against the Rfam database with default parameters using the Infernal software (Nawrocki and Eddy, 2013).

### 2.8 Structural variation identification in Hamlin sweet orange

Whole-genome synteny alignment between the CSH genome and the CSN genome was performed using the nucmer module integrated in MUMmer software (Marçais et al., 2018) to generate a standard delta alignment file. The parameters were set as follows: --threads 20 --mincluster 100 --breaklen 1000, to ensure the capture of long-range collinear signals across chromosomal-level scaffolds. Subsequently, Assemblytics was executed with a predefined variation size range from 50 bp to 100 kb, to comprehensively identify multiple categories of structural variations (SVs), including insertions and deletions (Indels), copy number variations (CNVs), translocations, and inversions, across the two genomes.

### 2.9 Identification and classification of MADS-box genes

Hidden Markov Models (HMMs) for the two core conserved domains of the MADS-box gene family, K-box (PF01486) and SRF-TF (PF00319), were obtained from the Pfam database. The HMMER software (Eddy, 2011) was used to mine homologous sequences in the genomes of CSN and CSH. The homologous sequences were annotated for domains using the NCBI CDD database (Marchler-Bauer et al., 2017). Sequences retaining complete MADS-box domains were identified as sweet orange MADS-box genes.

For classification, multiple sequence alignment of sweet orange and representative plant MADS-box sequences was performed using MAFFT v7.526 (Katoh and Standley, 2013) with the G-INS-i algorithm, followed by manual adjustment. A maximum likelihood phylogenetic tree was constructed using FastTree v2.2 (Price et al., 2010). Similarly, multiple sequence alignment and phylogenetic tree construction were performed for sweet orange and *Arabidopsis* MADS-box genes to classify the sweet orange MADS-box gene family members into subfamilies.

### 2.10 Characteristic analysis of sweet orange MADS-box genes

The physicochemical properties of the MADS proteins were determined using TBtools (Chen et al., 2020) by inputting the amino acid sequences. This analysis yielded results including amino acid number, relative molecular weight, isoelectric point, instability index, aliphatic index, and average hydrophobicity. The subcellular localization of MADS proteins was predicted using online tools CELLO, WoLF PSORT (Horton et al., 2007), and Cell-Ploc (Chou and Shen, 2008), and the results were compared to determine the most probable location.

Based on the GFF3 annotation files of the CSH and CSN genomes, the chromosomal localization of MADS-box genes was visualized using TBtools to reveal their distribution characteristics. The MEME online tool (Bailey et al., 2015) was used to analyze conserved motifs in protein sequences, with the maximum number of motifs set to 9. Finally, TBtools’ GeneStructureView (Advanced) function was utilized to integrate conserved motifs, conserved domains, and exon-intron information to construct a comprehensive analysis figure.

Promoter sequences (1500 bp upstream of the start codon) of each MADS-box gene were extracted and analyzed using the PlantCARE (Lescot, 2002) database to predict and annotate cis-acting elements, aiming to explore the potential transcriptional regulation mechanisms.

### 2.11 Transcriptome data analysis

Raw sequencing data were filtered using fastp (Chen et al., 2018) to remove reads containing adapters. The filtering criteria included removing paired reads if the content of N bases exceeded 10% or if low-quality bases (Q ≤ 20) accounted for more than 50% of the read. Subsequent analyses were based on the filtered clean reads. A reference genome index was built using HISAT2 (Kim et al., 2019), and clean reads were aligned to the reference genome.

New gene prediction was performed using StringTie (Pertea et al., 2015), which employs a network flow algorithm and optional *de novo* assembly strategy to assemble transcripts. Gene expression levels were quantified using featureCounts (Liao et al., 2013) to count mapped reads and calculate FPKM values based on gene length. Differential expression analysis between groups was performed using DESeq2 (Love et al., 2014), with P-values corrected using the Benjamini & Hochberg method (Wilcox, 2021). Significantly differentially expressed genes were screened using corrected P-values and |log2FoldChange| as thresholds. Functional enrichment analysis was performed based on a hypergeometric distribution test, with KEGG analysis conducted at the pathway level and GO analysis at the GO term level. Differential alternative splicing events were detected using rMATS (Shen et al., 2014), covering five main types: SE, RI, MXE, A5SS, and A3SS. SNP variant sites were detected using GATK (Chen and Madzima, 2026) and functionally annotated using ANNOVAR (Wang et al., 2010).

### 2.12 Quantitative real-time PCR analysis

Specific qPCR primers for 15 differentially expressed genes identified by transcriptome sequencing were designed using the SnapGene software (**Supplementary Table 1**). Total RNA was extracted and treated with DNase I to remove genomic DNA, followed by reverse transcription of 1 μg RNA into cDNA using the Takara PrimeScript™ 1st Strand cDNA Synthesis Kit. The cDNA was diluted 10-fold and used as a template for amplification on a LightCycler^®^ 480 real-time fluorescence quantitative PCR instrument. The 20 μL reaction system contained 10 μL 2×PerfectStart Green qPCR SuperMix, 0.4 μL forward primer, 0.4 μL reverse primer, 1 μL cDNA template, and ddH_2_O. The amplification program was set as follows: 95 °C for 1 min; 40 cycles of 95 °C for 5 sec and 60 °C for 30 sec; followed by a melting curve analysis (95 °C for 15 sec, 60 °C for 1 min, 95 °C for 15 sec). *CsGAPDH* was used as the internal control gene. The relative expression level was calculated using the 2^(-ΔΔCT) method (Schmittgen and Livak, 2008), with three biological replicates and three technical replicates set for each experiment to ensure data reliability.

### 2.13 Hormone extraction and quantification

For hormone extraction, 0.1 g of the sample was accurately weighed and thoroughly ground into a fine powder in liquid nitrogen. The powder was then transferred to a 2 mL centrifuge tube, and 1 mL of ethyl acetate extraction solvent was added. The tube was placed on a shaker and agitated at 2,000 rpm for 10 min, followed by centrifugation at 12,000 rpm for 10 min to collect the supernatant. The extraction step was repeated by adding 0.5 mL of the extraction solvent to the remaining pellet, and the supernatants from both rounds were combined. The combined extracts were dried under a stream of nitrogen and reconstituted in 200 μL of methanol. The solution was then filtered through a 0.22 μm membrane, and a 2 μL aliquot of the filtrate was injected for analysis.

Endogenous hormones, including GA1, GA, GA4, IAA, JA, SA, and cytokinins, were quantified using an ultra-high-performance liquid chromatography-tandem mass spectrometry (UPLC-MS/MS) system (AB SCIEX TripleTOF 5600+, Darmstadt, IN, USA). Chromatographic separation was achieved on an ACQUITY UPLC HSS T3 column (1.8 μm, 2.1 × 100 mm; Waters, USA) with acetonitrile as the mobile phase. The mass spectrometry parameters were set as follows: spray voltage, 4500 V; curtain gas, 15 psi; nebulizer gas, 65 psi; auxiliary gas, 70 psi; and source temperature, 400 °C. Each sample was analyzed with five biological replicates.

### 2.14 WGCNA analysis

WGCNA was performed using the WGCNA-shinyApp software (Langfelder and Horvath, 2008). Raw gene counts were first normalized using variance stabilizing transformation (VST) (Lin et al., 2008). A two-step gene filtering process was applied: genes with counts less than 10 in 90% of the samples were removed, followed by further selection of highly variable genes using the median absolute deviation (MAD) method (Pham-Gia and Hung, 2001). Based on the filtered data, the soft-thresholding power was calculated. The network was constructed with a minimum module size (minModuleSize) of 30 and a module cutting height (moduleCutHeight) of 0.25. Samples from stages where secondary fruit appeared were assigned a value of “1”, and those where it did not appear were assigned “0”. The correlation between modules and trait data was calculated to identify candidate genes based on significant “module-trait” associations. Finally, genes with both module membership (kME) and gene significance (GS) greater than 0.6, and edge weights higher than 0.8, were selected as Hub genes.

## 3 Results

### 3.1 *De novo* assembly and annotation of the Hamlin sweet orange genome

**Figure 1:**
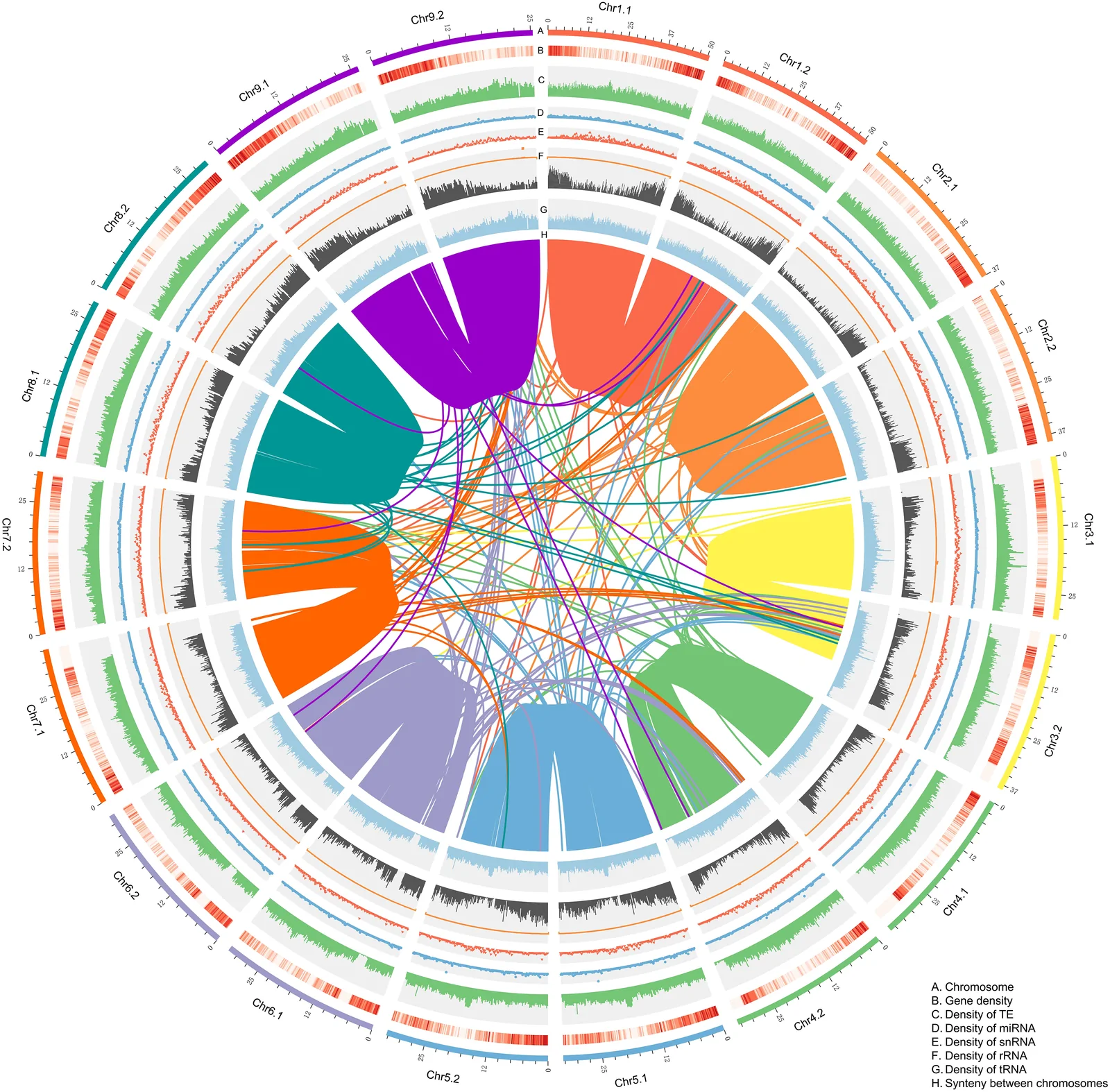
Genomic features of the Citrus sinensis ‘Hamlin’. **(A–G)** The circle graph depicts the genomic characteristics, including chromosome length **(A)**, gene density **(B)**, density of transposable element **(C)**, density of miRNA **(D)**, density of snRNA **(E)**, density of rRNA **(F)**, density of tRNA **(G)**, and the synteny between homologous chromosomes **(H)**.

In this study, we successfully completed the first high-quality *de novo* assembly of the CSH genome, yielding a highly continuous and complete reference genome at the chromosome level (**Figure 1**). The total assembled length was 673.06 Mb, which aligns closely with the classical diploid genome size of the genus Citrus (~680 Mb) and meets the expected genome size for *Citrus sinensis*. This indicates that the assembly is free from significant sequence loss or redundant amplification. Notably, the assembly exhibits exceptional continuity: the entire genome is covered by only 312 scaffolds with a mean length of 2.16 Mb and an impressive N50 of 32.89 Mb. This N50 value approaches the telomere-to-centromere scale, suggesting that the vast majority of scaffolds likely represent complete or nearly complete chromosome arms. Given that C. sinensis has a diploid chromosome number of 2n = 18, the theoretical maximum number of chromosome arms is approximately 36. Coupled with an extremely low total number of unknown bases (N; only 1,900 bp) and a minimal number of physical gaps (n = 19), these metrics confirm that our assembly achieves near-gapless, chromosome-arm-level continuity, making it with exceptionally high genomic continuity of citrus. The GC content was 37.19%, slightly higher than that of typical temperate fruit trees (apple at 36.4% and grape at 38.0%), reflecting the typical gene density and transposable element composition characteristic of the Rutaceae family (**Table 1**). The three independent methweods were applied to assess the genome completeness, and all the results confirmed that the assembled genome reached the level of a high-quality, excellent reference genome (**Supplementary Table 2**).

**Table 1:** Genome information of two sweet orange varieties.

Assembly metrics	Genome of *Citrus sinensis* ‘Hamlin’	Genome of *Citrus sinensis* ‘Newhall’
Total length(bp)	697,063,978	685,274,098
Scaffold Number	479	1,013
Average length(bp)	1,455,454	676,480
N50(bp)	32,891,559	32,860,738
N90(bp)	25,366,080	25,721,200
Number of unknown bases (N)	1,900	61,100
Number of gaps	19	611
GC content	37.19%	36.84%
BUSCO	C:96.95%[S:96.8%, D:0.15%], F:1.1%, M:1.95%, n:1614	C:96.95%[S:95.1%, D:9.3%]*, F:1.5%, M:3.4%, n:1440

*: data form Gao et al., 2023.

A comparative analysis between the CSN and CSH genomes revealed that although the CSN genome is marginally longer (+1.8%)(**Table 1**). it consists of 1,013 scaffolds (3.25 times more than CSH) with a mean length of only 676.5 kb (less than one-third of CSH)(**Table 1**). Furthermore, the CSN assembly contains 611 gaps, which is 32 times higher than that of CSH (**Table 1**). Remarkably, the CSH assembly exhibits a 96.9% reduction in both unknown bases (N) and gaps. The high consistency between the N50 and N90 values (difference < 0.1%) indicates comparable completeness in the core gene space of both cultivars, confirming that coding genes, promoters, and proximal regulatory regions have been effectively captured.

To further elucidate the genomic-level differences between CSN and CSH sweet orange, we conducted a systematic comparative analysis of the assembly quality and SVs of both genomes. Genome assembly evaluation (**Supplementary Figure 1**) demonstrated high continuity and completeness for both versions. The cumulative sequence length plot showed a steady decline as NG(x)% increased, indicating an absence of massive fragmented short sequences. The assembly quality was excellent, with an N50 of contig reaching 32.89Mb and 5 complete chromosomes (**Supplementary Table 3**), providing a reliable foundation for subsequent SVs detection.

Regarding structural variations, we identified a total of 131 significant SV events (**Supplementary Figure 2**). Tandem repeat expansion was the predominant type of variation (n = 42), followed by insertions (n = 36) and deletions (n = 26). This distribution suggests that the amplification of tandem repeats may be a primary driving force in genome evolution and phenotypic divergence between the two sweet orange cultivars. Size distribution analysis of SVs ranging from 500 bp to 10,000 bp (**Supplementary Figure 3**) revealed type-specific length patterns. Insertions and deletions were primarily concentrated in smaller size ranges, whereas some tandem repeat expansion events were relatively large (approaching 7,000–8,000 bp), potentially corresponding to copy number variations of specific functional gene families or regulatory elements. Chromosome-level distribution analysis (**Supplementary Figure 4**) highlighted a non-uniform distribution of SVs. Chromosome CSN_Chr5_1 was enriched with the highest number of SVs (n = 24), followed by CSN_Chr7_2 (n = 13) and CSN_Chr1_1 (n = 10). In contrast, only two SVs were detected on other chromosomes, such as CSN_Chr9_13. This chromosome-specific hotspot distribution may indicate that these regions have undergone more intense genomic remodeling during the adaptive evolution or the formation of key traits (disease resistance and fruit quality) in Newhall navel orange and Hamlin sweet orange.

### 3.2 Genome-wide identification of sweet orange MADS-box genes

**Figure 2:**
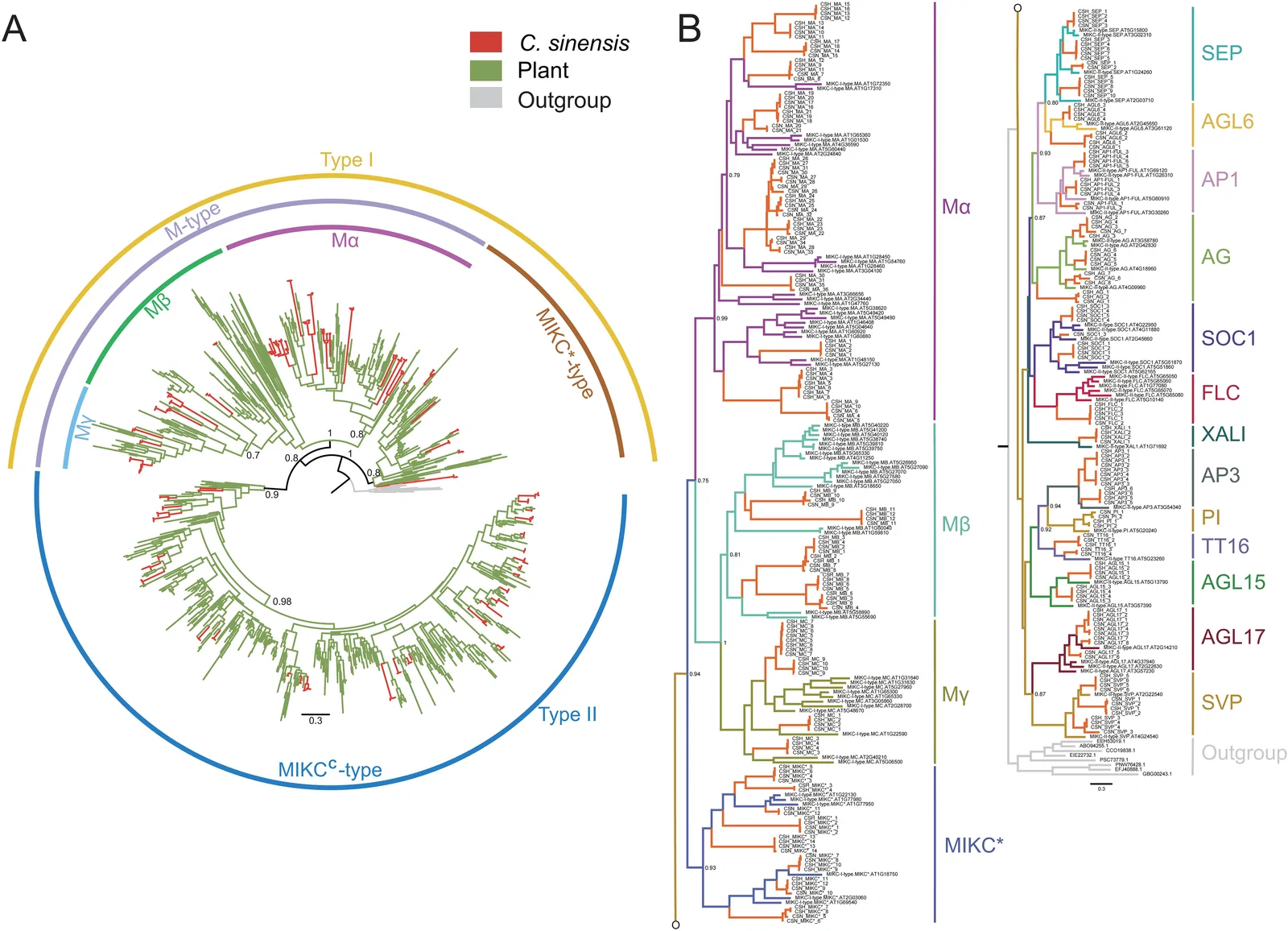
Phylogenetic relationships of sweet orange MADS-box proteins. **(A)** Phylogenetic tree constructed using MADS-box protein sequences from sweet orange and published plant species. The tree was built using the maximum likelihood method, with sequences from chlorophytes used as the outgroup to root the tree. Protein sequences from different species are distinguished by terminal node colors: red represents sweet orange (C. sinensis) proteins, green represents proteins from other plant species, and gray represents the outgroup. Based on domain characteristics and evolutionary relationships, the MADS-box family is divided into two major types (Type I and Type II), further subdivided into multiple subfamilies. Numbers on the branches indicate likelihood values and only the key branches with a value greater than 0.5 are marked. **(B)** Phylogenetic tree constructed using MADS-box protein sequences from sweet orange and Arabidopsis thaliana. This tree was also built using the maximum likelihood method, rooted with sequences from chlorophytes as the outgroup. Branches are divided into four major clades based on evolutionary relationships: MA, MB, MC, and MIKC*. The MIKC* clade is further subdivided into functional subfamilies such as SEP, AGL6, AP1, AG, SOC1, FLC, XALI, AP3, PI, TT16, AGL15, AGL17, and SVP (annotated by colored labels on the right). Sequences from ‘Newhall’ navel orange contain the prefix ‘CSN’, while sequences from ‘Hamlin’ sweet orange contain the prefix ‘CSH’. Numbers on the branches indicate likelihood values and only the key branches with a value greater than 0.5 are marked.

Using comparative genomics, we identified 139 and 119 MADS-box proteins in the proteomes of Newhall navel orange and Hamlin sweet orange, respectively. The copy number of each category of MADS-box genes varies across genomes. Specifically, the MA subfamily contains 32 copies in CSH and 36 copies in CSN, respectively. The AG gene, which performs critical regulatory functions, also exhibits distinct copy numbers between these two Citrus species (**Supplementary Table 4**). Phylogenetic analysis of these proteins alongside published plant MADS-box sequences revealed that sweet orange MADS-box genes can be classified according to the established MADS gene family classification system (**Figure 2A**). The family is primarily divided into Type I and Type II. Type I includes multiple subclasses, such as the M-type, with sweet orange MADS-box genes distributed across various clades within this group, demonstrating their diversity (**Figure 2A**). Type II is further subdivided into MIKC*-type and MIKC^C^-type. Sweet orange MADS-box genes are also broadly distributed within Type II, forming complex evolutionary clades with homologous genes from other plants. The widespread dispersion of sweet orange MADS-box genes across multiple branches on the phylogenetic tree indicates rich diversity, suggesting functional differentiation among clades; some may be associated with floral organ development, while others may participate in fruit development and stress responses.

To explore the potential functions of sweet orange MADS-box proteins, we constructed a phylogenetic tree using sequences with known functions from *Arabidopsis thaliana* (**Figure 2B**). The analysis clearly partitioned the sweet orange MADS-box protein family into multiple evolutionary clades, with a topology highly consistent with the functional classification of *Arabidopsis* homologs. This reveals the conservation and functional divergence of this transcription factor family throughout plant evolution. Sweet orange MADS-box proteins primarily clustered into the MA, MB, MC, and MIKC major clades. The MIKC clade was further subdivided into functional subfamilies, including SEP, AGL6, AP1, AG, SOC1, FLC, XALI, AP3, PI, TT16, AGL15, AGL17, and SVP (**Figure 2B**). Notably, sweet orange proteins have corresponding orthologs in all subfamilies. For instance, sweet orange proteins in the SEP subfamily form tight clusters with *Arabidopsis* SEP1-4 (**Figure 2B**), supporting their conserved function in floral organ identity determination. Similarly, sweet orange homologs in the AG subfamily cluster with *Arabidopsis* AG (**Figure 2B**), implying their potential roles in stamen and carpel development. Additionally, some sweet orange proteins are located in the FLC and SVP clades, suggesting their potential involvement in flowering time regulation (**Figure 2B**). Comparing the subfamily distribution of the MADS-box gene family between the two species showed that most subfamilies (MIKC, MB, MC, AP3, AGL, SVP, etc.) maintained highly consistent gene numbers (**Supplementary Figure 5**). Nevertheless, interspecific differences were found in some subfamilies, where ‘Newhall’ navel orange had slightly more genes in the SEP, AP1-FUL, AGL17, SOC1, and TT16 subfamilies compared to ‘Hamlin’ sweet orange (**Supplementary Figure 5**).

Bioinformatic analyses were conducted to predict the physicochemical properties and subcellular localization of the MADS-box transcription factor family in CSH and CSN. The 258 identified MADS-box genes exhibited significant diversity in amino acid length, molecular weight, and isoelectric point (pI). The theoretical pI of most proteins was alkaline (> 7.0), and instability index analysis indicated that the majority are stable (**Supplementary Table 5**). Subcellular localization predictions (Yu et al., 2006) consistently showed that most MADS-box proteins are localized in the nucleus, aligning with their biological function as transcription factors regulating downstream gene expression (**Supplementary Table 5**). Notably, some genes were predicted to be localized in the chloroplast, cytoplasm, or mitochondrion, suggesting potentially broader and more diverse regulatory roles during citrus growth and development.

### 3.3 Characterization of sweet orange MADS-box genes

**Figure 3:**
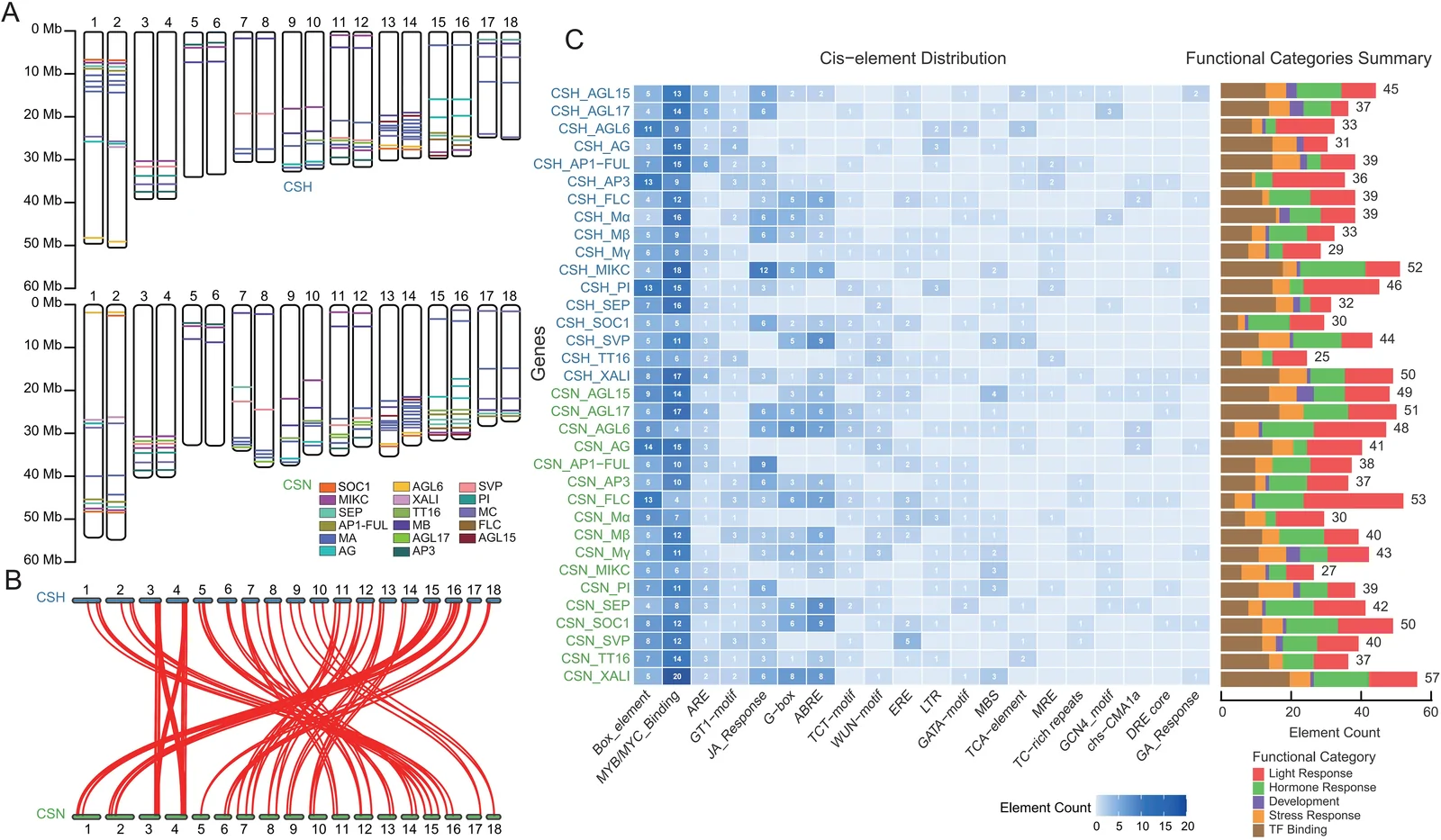
Analysis of sweet orange MADS-box genes. **(A)** Physical localization of MADS-box genes on the chromosomes of ‘Newhall’ navel orange and ‘Hamlin’ sweet orange. The figure displays the positions of the sweet orange MADS-box gene family across 18 chromosomes. The upper half represents the gene distribution in ‘Hamlin’, while the lower half represents that in ‘Newhall’. Different colors denote different MADS-box subfamilies (as shown in the legend), and the positions of genes on the chromosomes are marked by their physical coordinates (Mb). **(B)** Synteny analysis of MADS-box genes between the two sets of chromosomes. This panel illustrates the syntenic relationships of MADS-box genes between the two chromosome sets (CSH and CSN). The red lines **(synteny links)** indicate MADS-box gene pairs with significant homology between the two chromosome sets. The density and distribution pattern of these links reflect the evolutionary conservation and rearrangement events of the genomic regions. **(C)** Cis-acting element analysis of MADS-box genes in CSH and CSN. Gene colors represent the species of origin, with blue indicating ‘Hamlin’ and green indicating ‘Newhall’.

To elucidate the genomic organization of the sweet orange MADS-box gene family, we mapped the identified genes onto the 18 chromosomes of CSN and CSH (**Figure 3A**). The results revealed a significantly uneven genome-wide distribution: certain chromosomes (Chr1 and Chr2) exhibited distinct gene enrichment, while other regions were gene-poor. The two cultivars exhibited high synteny and structural conservation; however, individual gene deletions or translocations were observed in specific regions, such as the upper part of Chr1 and the lower part of Chr2. These structural variations may be closely associated with their phenotypic differences.

Synteny analysis between the two genomes further revealed the evolutionary conservation of MADS-box genes (**Figure 3B**). Highly concentrated synteny links in regions of Chr3, 4, 13, 14, 17, and 18 indicate that these regions (potentially containing floral development-related gene clusters) have maintained extremely high gene order conservation during evolution. Conversely, sparse or crossed links in regions of Chr1, 5, and 6 suggest that these chromosomal segments may have undergone structural variation events such as inversions, translocations, or gene loss.

Promoter *cis*-acting element analysis showed similar overall distribution patterns between the two cultivars, but significant differences in the enrichment of specific elements (**Figure 3C**). CSN exhibited a higher enrichment of light-responsive elements in genes such as *AGL15* and *SVP*, and significantly more stress-responsive elements (MBS, LTR) in the Type I family (MA, MB) compared to CSH. This implies that CSN may be more sensitive to light signals and possesses greater regulatory potential in abiotic stress responses (drought and low temperature). In contrast, both cultivars showed comparable enrichment of development-related elements in core floral development genes such as *SEP* and *AG*, indicating high conservation of their fundamental functions in floral organ formation. There is no significant difference in *cis*-acting element among the duplicated copies generated by gene duplication events (**Figure 3C**). These differences in *cis*-regulatory networks provide a molecular basis for understanding the phenotypic divergence in fruit development and environmental adaptability between the two cultivars.

To further investigate the evolutionary characteristics and functional conservation of the MADS-box transcription factor family in CSH and CSN, we conducted systematic motif, gene structure, and conserved domain analyses. Both cultivars contained highly conserved motif combinations; the vast majority of members possessed Motif 1 and Motif 2, corresponding to the core MADS domain sequence, confirming their classification as typical MADS-box genes (**Supplementary Figure 6**, **7**). Gene structure comparison revealed a coexistence of diversity and conservation in intron-exon patterns. Most MADS genes contained multiple introns (gray lines) and a relatively fixed number of exons (colored boxes), consistent with general plant MADS-box gene characteristics (**Supplementary Figure 6**, **7**). There is no significant difference in gene structure among the duplicated copies generated by gene duplication events. However, some genes exhibited significant structural variations. For example, certain *SVP* or *AGL* homologs contained notably more introns or exceptionally long intronic regions (*CSH_SVP_1* and *CSN_SVP_1*). These structural differences may stem from intron insertion/loss events or transposable element activity during evolution, providing a structural basis for functional diversification. Notably, despite length variations, genes within the same subfamily generally maintained consistent exon phases and relative positions, further supporting their common origin.

Scans using Pfam and SMART databases detected the core MADS domain in all identified proteins, which is critical for DNA binding and dimerization (**Supplementary Figure 6**, **7**). Additionally, based on the ABC model classification, we observed K-box domains (yellow bars) and C-terminal functional domains. MIKC-type genes (*AP1*, *AP3*, *PI*, *AG*, *SEP*) universally possessed a complete MADS-K-C architecture, where the K-box mediates protein-protein interactions and the C-terminal domain is responsible for transcriptional activation or repression. For α/Mβ-type genes (*SOC1* or *SVP* homologs), some members may lack typical K-boxes, possess incomplete C-terminal domains, or contain specific Mα/Mβ subtype characteristic domains, suggesting that their regulatory mechanisms may differ from classical MIKC-type proteins.

### 3.4 Transcriptomic analysis and hormone profiling of secondary fruit development in Newhall navel orange

**Figure 4:**
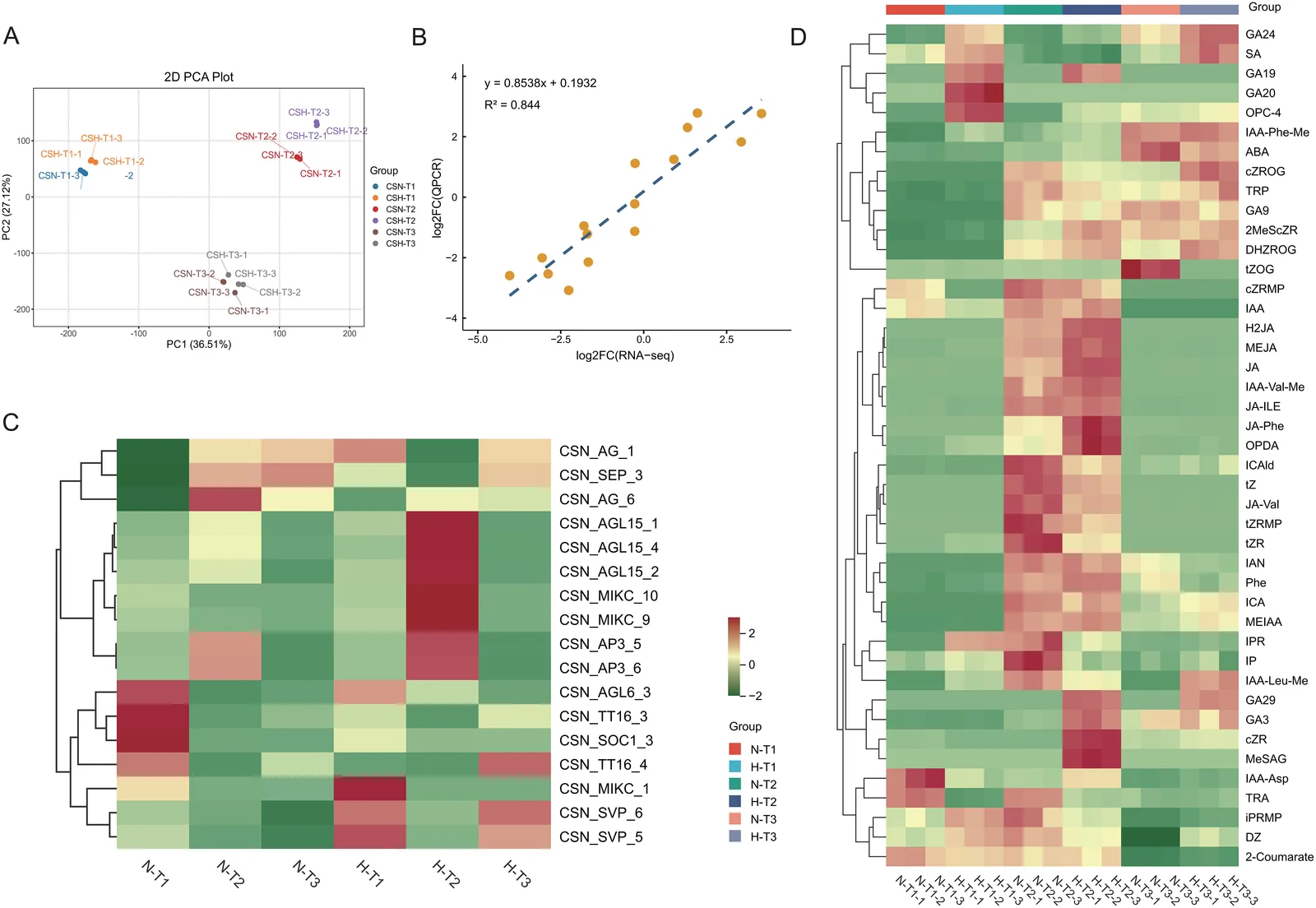
Transcriptomic and Hormone profiling and validation of *MADS-box* genes expression in sweet orange varieties. **(A)** Principal Component Analysis (PCA) of transcriptome samples. The 2D PCA plot illustrates the distribution of biological replicates for CSN and CSH across three time points (T1, T2, T3). PC1 and PC2 explain 36.51% and 27.12% of the total variance, respectively. Distinct clustering is observed among different time points, indicating significant transcriptional changes during development, while biological replicates within each group show high consistency. **(B)** Validation of RNA-seq data by qRT-PCR. Scatter plot comparing the log2 fold change (log2FC) values obtained from RNA-seq **(x-axis)** and qRT-PCR **(y-axis)** for selected genes. The strong positive correlation (*R^2^* = 0.844) indicates that the RNA-seq results are reliable and accurate. **(C)** Heatmap of expression patterns for selected MADS-box genes. Hierarchical clustering of specific MADS-box genes across different samples. The color scale represents normalized expression levels (red indicates high expression, green indicates low expression). Distinct expression clusters are observed across different developmental stages (T1-T3), suggesting their potential roles in regulating fruit development or variety-specific traits. **(D)** Hierarchical clustering heatmap of quantified phytohormone metabolites. Rows represent 47 detected hormone-related compounds, including gibberellins (GA), auxins (IAA), cytokinins (tZR, cZR and derivatives), jasmonates (JA, MEJA and derivatives), abscisic acid (ABA) and other signaling molecules. columns represent three biological replicates of two citrus cultivars across three flower developmental stages. The color gradient from green to red indicates normalized low to high metabolite abundance, showing distinct cultivar- and stage-specific accumulation patterns of key phytohormones.

To elucidate the developmental mechanism of secondary fruits in CSN, we performed transcriptomic sequencing on floral samples from CSN and CSH at the bud stage (T1), full bloom stage (T2), and post-bloom stage (T3). Principal component analysis (PCA) (**Figure 4A**) showed that PC1 and PC2 explained 36.51% and 27.12% of the total variance, respectively. Samples exhibited significant temporal separation along the PC1 axis (arranged sequentially from T1 to T2 to T3), indicating that the developmental process drives the continuous evolution of gene expression patterns. Along the PC2 axis, samples of CSH and CSN from the same period (especially at T2 and T3) showed vertical separation, revealing cultivar-specific gene expression regulatory patterns. Furthermore, biological replicates (N-T1-1/2/3) clustered tightly, confirming excellent reproducibility and providing a solid foundation for subsequent differential expression analysis.

Pearson correlation coefficient analysis further validated the reliability of the transcriptomic data and the consistency of biological replicates (**Supplementary Figure 8**). The correlation matrix demonstrated extremely high intra-group correlation (r > 0.96); for instance, the correlation coefficient among CSN T1 replicates reached 0.98–0.99, and 0.99 for CSH T2, confirming the high stability of the RNA-seq experiments. Inter-group comparisons showed that the correlation between different developmental stages of the same cultivar (r ≈ 0.81–0.92) was slightly lower than intra-group replicates, reflecting the gradual evolution of gene expression profiles during development. Meanwhile, the relatively lower correlation between different cultivars at the same stage (r ≈ 0.77–0.85) further corroborated significant cultivar-specific differences at the transcriptional level. Collectively, these correlation analyses are highly consistent with the PCA results, fully demonstrating the high quality of the dataset and its suitability for subsequent differential gene screening and functional enrichment analysis.

To verify the accuracy and reliability of the RNA-seq data, we randomly selected differentially expressed genes (DEGs) for validation using quantitative real-time PCR (qRT-PCR). By calculating the log2 fold change (log2FC) values for each gene under both detection methods and plotting a scatter plot for correlation analysis (**Supplementary Table 6**), the data points exhibited a clear linear trend, clustering tightly around the fitted line (R² = 0.844) (**Figure 4B**). This indicates a significant positive correlation and highly consistent trends between qRT-PCR and RNA-seq results. Despite minor discrepancies in absolute expression levels for individual genes, the overall concordance strongly validates the authenticity and reliability of the RNA-seq data, confirming that the sequencing results accurately reflect the relative changes in gene expression among samples.

To explore the transcriptional regulatory differences of MADS-box family genes between CSH and CSN across the three developmental stages (T1, T2, T3), we performed hierarchical clustering heatmap analysis on the screened DEGs (fold change > 2) (**Figure 4C**). First, genes specifically highly expressed in CSN at T1 and T2, such as *CSN_AG_1*, *CSN_TT16_3*, *CSN_SEP_3*, and *CSN_AGL_3*, may be associated with secondary fruit development and warrant focused attention. Additionally, some genes exhibited strong cultivar specificity. For example, *CSN_AGL15_1*, *CSN_AGL15_2*, and *CSN_MIKC_9* were expressed at extremely high levels in CSH at T2, but at very low levels in CSN and other stages of CSH (**Figure 4C**). Second, some genes displayed dynamic trends changing with developmental progression. For instance, *CSN_SOC1_3* and *CSN_SVP_6* showed relatively high expression levels at T3 in both cultivars, suggesting they may be jointly involved in regulating fruit ripening or late-stage floral organ development. Overall, this heatmap reveals the complex regulatory network of the MADS-box gene family across different developmental stages in the two sweet orange cultivars, providing crucial candidate gene clues for elucidating the molecular mechanisms of floral development and fruit formation.

To elucidate the differences in hormonal regulation during flower development between the two cultivars, we performed targeted phytohormone profiling on flowers at three developmental stages, successfully quantifying the levels of multiple endogenous hormones (**Supplementary Table 7**). The active gibberellin (GAs), exhibited a sharp increase at the full-bloom stage and remained at a high level during the post-bloom stage in both CSN and CSH. Conversely, abscisic acid (ABA) accumulated continuously in both cultivars, peaking at the post-bloom stage, which may be associated with the senescence process of floral organs. Different forms of cytokinins showed functional differentiation: the transport-active form, Cytokinins (tZR), peaked at full bloom and then declined rapidly, whereas the storage form, (tZOG), accumulated extensively at the post-bloom stage (**Figure 4D**).

During the full-bloom stage, a critical period determining fruit set, the two cultivars exhibited significant differences in their hormonal profiles (**Figure 4D**). The GA content in CSH was 12.74 ng/g, significantly higher than that in CSH (3.55 ng/g) (p < 0.01; **Supplementary Table 7**). As GAs are key hormones promoting cell elongation and fruit set, this difference aligns perfectly with the normal fruit set and seeded fruit development observed in CSH. The tZR content in CSH was 49.93 ng/g, significantly higher than that in CSH (20.97 ng/g) (p < 0.01; **Supplementary Table 7**). As an active signaling molecule promoting cell division, the massive accumulation of tZR in CSH at full bloom may directly drive the excessive proliferation of meristematic tissues at the receptacle, laying the physiological foundation for the formation of secondary fruits. Although no significant difference in Auxins (IAA) content was observed between the two cultivars at the full-bloom stage (22.84 ng/g in CSH vs. 22.52 ng/g in CSH), CSN maintained a higher basal IAA level during the bud stage, potentially providing the initial driving force for subsequent organogenesis. While showing no significant difference at full bloom, the ABA content in CSN was significantly higher than that in CSH at the post-bloom stage, suggesting that its senescence or stress-response processes may proceed more rapidly.

Additionally, to evaluate the relative strength of growth-promoting signals versus stress/senescence signals, we calculated the GAs/ABA and tZR/ABA ratios at the full-bloom stage for both cultivars. The ratio of GAs/ABA in CSH (0.035) was significantly higher than that in CSN(0.012) (p < 0.01; **Supplementary Table 7**). This indicates that despite similar ABA levels, the GAs signal in CSH overwhelmingly dominates over the stress signal, which is conducive to cell elongation and the initiation of fruit set. The ratio of tZR/ABA in CSN (0.17) was significantly higher than that in CSH (0.057) (p < 0.01; **Supplementary Table 7**). This further corroborates that, under a similar ABA background, the cell division signal in CSN has a massive advantage over the stress signal, facilitating the maintenance of meristematic activity and establishing the foundation for secondary fruit formation.

### 3.5 WGCNA identifies key regulatory genes for secondary fruit development in Newhall navel orange

**Figure 5:**
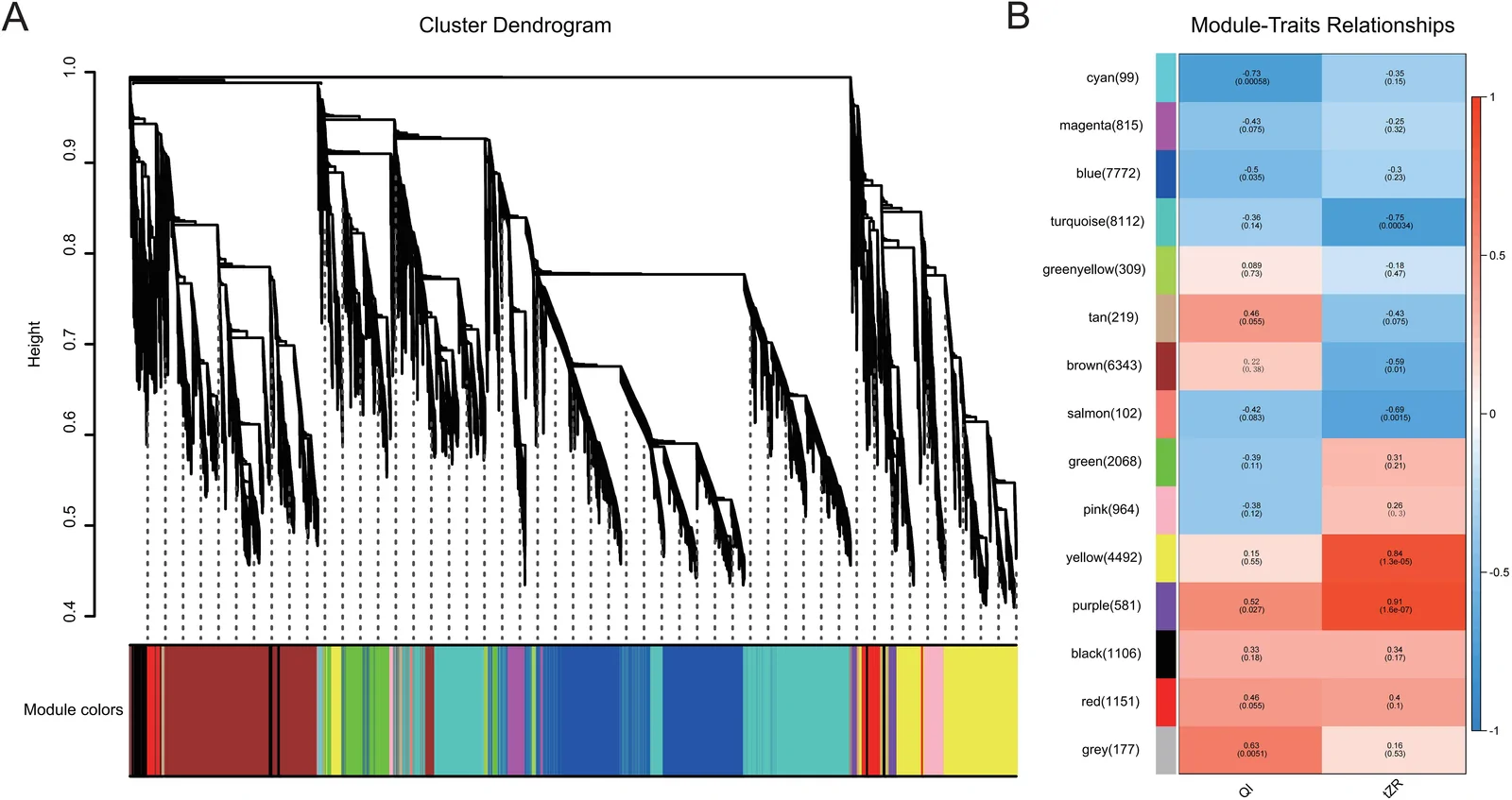
Weighted gene co-expression network analysis (WGCNA) and module-trait relationship analysis. **(A)** Gene Clustering Dendrogram. Hierarchical clustering results based on the Topological Overlap Matrix (TOM), displaying the clustering relationships of all genes. The colored bars at the bottom represent the distinct co-expression modules identified by the dynamic tree-cutting algorithm, with each color corresponding to a specific gene module. **(B)** Module-Trait Relationship Heatmap. This panel illustrates the Pearson correlation coefficients between each co-expression module and specific traits (columns). The color intensity of the blocks indicates the strength of the correlation (red represents positive correlation, blue represents negative correlation). The values within the blocks denote the correlation coefficients and their corresponding P-values (in parentheses).

To explore the intrinsic relationship between gene expression patterns and specific biological traits, we systematically analyzed the transcriptomic data using Weighted Gene Co-expression Network Analysis (WGCNA). Soft threshold screening analysis using the WGCNA package revealed that the scale-free topology fit index first exceeded 0.8 when the power reached 12. Therefore, β = 12 was selected as the soft threshold parameter for constructing the co-expression network (**Supplementary Figure 9**). Based on the selected β value, the Topological Overlap Matrix (TOM) was calculated, and hierarchical clustering was performed using the dissimilarity measure (1-TOM) (**Supplementary Figure 10**). Gene modules were identified via dynamic tree cutting, and modules with highly similar expression patterns were merged. Ultimately, 15 gene co-expression modules were identified (**Figure 5A**). Based on the module-trait relationship heatmap (**Figure 5B**; **Supplementary Figure 11**), the purple module exhibited significant positive correlations with the target trait. Analysis revealed that samples of CSN at the T2 stage were significantly upregulated in the purple module (**Supplementary Figure 12**). To identify hub genes that play a central regulatory role within the module, this study screened for hub genes from the purple module, which was significantly positively correlated with secondary fruit traits. The screening thresholds were set as Module Membership (MM) > 0.6 and Gene Significance (GS) > 0.6. Specifically, the MM value reflects the connectivity (centrality) of a gene within the module, while the GS value indicates the strength of the correlation between gene expression and the target trait (**Supplementary Figure 14A**).

The purple module was positively correlated with secondary fruit development, and 10 hub genes were successfully identified. GO annotation indicated that the biological processes of genes in this module included cell division and protein folding (**Supplementary Figure 14B**). Among these hub genes, two MADS-box genes were identified: *CSN_SEP_3* (evm.model.chr3.1.2090) and *CSN_AG_6* (evm.model.chr8.1.2342). *SEP* and *AG* are classic ABC model genes involved in determining floral organ identity and the development of carpels and stamens, respectively. Therefore, *CSN_SEP_3* and *CSN_AG_6* were identified as key candidate regulatory genes for secondary fruit development in CSN, necessitating further molecular validation in future studies.

Additionally, to investigate whether SVs between Newhall and Hamlin directly affect the expression of key candidate genes, we performed an overlap analysis between the genomic coordinates of 131 SVs and the genomic locations of all 10 hub genes in the purple module. The results revealed that none of the SVs were located within 5 kb upstream or downstream of these 10 hub genes. These findings suggest that the differential expression of hub genes in the purple module between Newhall and Hamlin is unlikely to be directly caused by large-scale structural variations in the coding or flanking regions. Instead, the regulatory differences in their expression may stem from more subtle sequence variations.

## 4 Discussion

Previous studies have suggested that the development of secondary fruits in navel oranges is driven by the overexpression of carpel identity genes. In the classical Arabidopsis model, a feedback loop between WUSCHEL (WUS) and CLAVATA3 (CLV3) maintains stem cell homeostasis during the early stages of floral development. During the mid-to-late stages, AG represses WUS expression and terminates stem cell activity, ultimately ensuring carpel formation and the completion of floral development (Liu et al., 2011). Our hormonal profiling data indicate that Newhall navel orange specifically accumulates exceptionally high levels of the active cytokinin tZR during the full bloom stage (Finure 4D). Furthermore, while AG is highly expressed during the full bloom stage in Newhall, WUS still exhibits residual expression. In contrast, WUS is completely silenced in Hamlin sweet orange (**Supplementary Figure 15**, **Supplementary Table 6**). We hypothesize that the elevated cytokinin levels in Newhall may partially interfere with the AG-mediated repression of WUS via the purple module, thereby creating an expression window where AG and WUS coexist. This may prolong meristem activity and subsequently participate in the induction of secondary fruits. Conversely, in Hamlin, the absence of high cytokinin levels may allow AG to normally repress WUS. This finding suggests that the regulatory mechanism of floral meristem termination in citrus may resemble that of Arabidopsis, although this requires further validation through exogenous cytokinin treatments and gene editing approaches. Notably, such a regulatory pattern is rarely reported in woody fruit trees.

The downregulation of *SOC1/SVP/AGL17*-like genes in the blue module may relieve the negative regulation on the *AG/SEP* pathway, thereby permitting carpel over-proliferation. This “release of repression” model has been previously reported (Lee et al., 2008; Busatto and Herrera, 2025). Future studies should conduct comprehensive molecular biological validations targeting all MADS-box genes within the purple and blue modules, as well as their downstream targets.

In this study, we successfully completed the first high-quality, chromosome-level *de novo* genome assembly of Hamlin sweet orange. Its continuity metrics (N50 = 32.89 Mb with only 19 gaps) represent the highest level of citrus genome assembly achieved to date (Hong et al., 2024). This assembly has essentially achieved chromosome-arm-level continuity. A direct comparison with the CSN genome (revealing a 68.4% reduction in scaffolds and a 96.9% reduction in gaps) further confirms that our assembly provides an unprecedentedly complete framework for subsequent SV detection, gene cluster resolution, and epigenetic studies.

At the structural variation level, we identified a total of 131 SV events, with tandem repeat expansions accounting for the highest proportion (42 events, 32.1%). This suggests that tandem repeat-driven gene family expansion may be a crucial evolutionary driving force in the varietal divergence between CSN and CSH. This pattern is consistent with studies in crops such as grape (Zhou et al., 2019), cacao (Motamayor et al., 2002), and soybean (Lam et al., 2010), where tandem repeats were found to be the predominant SV type. Our findings further extend this understanding to the mechanisms underlying rapid phenotypic differentiation within the asexual reproduction system of citrus. Chromosome Chr5_1 was enriched with 24 SVs (18.3% of the total). This hotspot distribution highly overlaps with known quantitative trait loci (QTLs) for fruit quality and disease-resistance NLR gene clusters (Wang et al., 2021), implying that this chromosome has undergone intense genomic remodeling. Similarly, the SV clustering detected on Chr7_2 and Chr1_1 aligns with the structural variation hotspots and genomic remodeling characteristics recently identified in sweet orange bud mutation populations based on high-quality genomes (Wang et al., 2021).

Although 131SVs were detected across both genomes, no SVs were identified near any key candidate genes involved in the secondary fruit development of Newhall navel oranges screened in this study; this suggests that these large structural variations likely arose directly within or adjacent to the coding regions, while any differential expression patterns may stem from more subtle sequence-level variations.

This study represents the first systematic identification of the MADS-box transcription factor family at the chromosome level in the genomes of CSN and CSH, yielding 139 and 119 members, respectively. The quantitative difference between the two cultivars (+20 genes) carries significant biological implications: CSN may have undergone local expansion events of the MADS-box gene family during asexual reproduction (bud mutation), which could be closely associated with its unique seedless phenotype and fruit morphological differentiation. Comparatively, the number of MADS-box genes varies substantially among species, with 94 genes in *Lagerstroemia suprareticulata*, 83 in *L. speciosa*, and 79 in *L. indica* (Wan et al., 2025), 75 genes in *Nephelium lappaceum* L (Dong et al., 2024), and up to 325 genes in *Camelina sativa* L (Tahmasebi et al., 2025).

The uneven distribution of MADS-box genes in the sweet orange genome is consistent with observations in most reported plants. In *Arabidopsis*, MADS-box genes tend to cluster in pericentromeric regions and gene-rich areas (Parenicova, 2003). In our study, Type I subfamily genes (MA and MB) formed tandem repeat clusters on Chr1 and Chr2, indicating that gene duplication is the primary mechanism for the expansion of the Type I family. This aligns with findings in cultivated alfalfa (*Medicago sativa* L.), where M-type MADS-box genes are massively amplified via tandem duplication (Dong et al., 2021). In contrast, Type II genes are more dispersed, with only localized gene clusters, which is consistent with their evolutionary characteristics as core nodes in transcriptional regulatory networks subject to stronger purifying selection (Becker, 2003). Our study systematically confirms the evolutionary model of Type I subfamily expansion via tandem duplication at the chromosomal level, further supporting the theory that “Type I genes in the MADS-box family evolve under a birth-and-death model, whereas Type II genes are subject to strong purifying selection” (Nam et al., 2004).

Through systematic quality control of transcriptomic data and weighted gene co-expression network analysis (WGCNA), this study provides a reliable data foundation and candidate genes for elucidating the molecular regulatory mechanisms of secondary fruit development in CSN. Pearson correlation coefficient analysis (intra-group *r* > 0.96) and qRT-PCR validation (*R*² = 0.844) further confirm the high quality of the dataset. This multi-dimensional validation strategy significantly enhances the credibility of subsequent differential expression and co-expression network analyses. Hierarchical clustering heatmaps revealed that MADS-box family genes exhibit significant cultivar-specific and developmental stage-dependent expression patterns between CSN and CSH. The specific high expression of *CSN_AG_1*, *CSN_TT16_3*, *CSN_SEP_3*, and *CSN_AGL_3* in CSN at the T1/T2 stages is highly consistent with the core functions of *AG*- and *SEP*-like genes in the classical ABC model (Theißen and Saedler, 2001). Notably, the upregulation of *AG*-like genes may be closely related to the morphological process of secondary fruit (navel) formation originating from carpel over-proliferation (Liu et al., 2011). WGCNA further specified this regulatory network by identifying 15 co-expression modules. The purple module (*r* = 0.52, *p* = 0.027) showed a significant positive correlation with secondary fruit development and was enriched with two MADS-box genes, *CSN_SEP_3* and *CSN_AG_6*. The blue module (*r* = -0.50, *p* = 0.035) exhibited a significant negative correlation and was enriched with 10 MADS genes, including *SOC1*, *SVP*, *AGL17*, *AGL6*, and *SEP_10*.

## 5 Conclusion

In this study, we *de novo* assembled and annotated a chromosome-level genome for ‘Hamlin’ sweet orange, and systematically characterized the identification and gene structural features of MADS-box genes in both ‘Hamlin’ sweet orange and ‘Newhall’ navel orange. By integrating transcriptomic and hormonal profiling, we identified key MADS-box genes involved in the development of secondary fruits in ‘Newhall’ navel orange. However, the underlying regulatory mechanisms of these MADS-box genes warrant further investigation in future studies.

## Data Availability

The assembled genome with annotations for Hamlin sweet orange generated in this study, as well as the raw RNA-seq and phytohormone data for both Hamlin sweet orange and Newhall navel orange across three flowering stages, are accessible in the Science Data Bank (https://www.scidb.cn/s/ERrIZj).
