# Supplementary material for: Chromosome-level genome assembly of Hamlin sweet orange and transcriptomic insights into MADS-box gene regulation of secondary fruit development
Source: Front Plant Sci. 2026 Sep 8;17:1925055. doi: 10.3389/fpls.2026.1925055 (PMC13593871; doi:10.3389/fpls.2026.1925055)
Supplement: Supplementary file 1 [file DataSheet1.pdf]

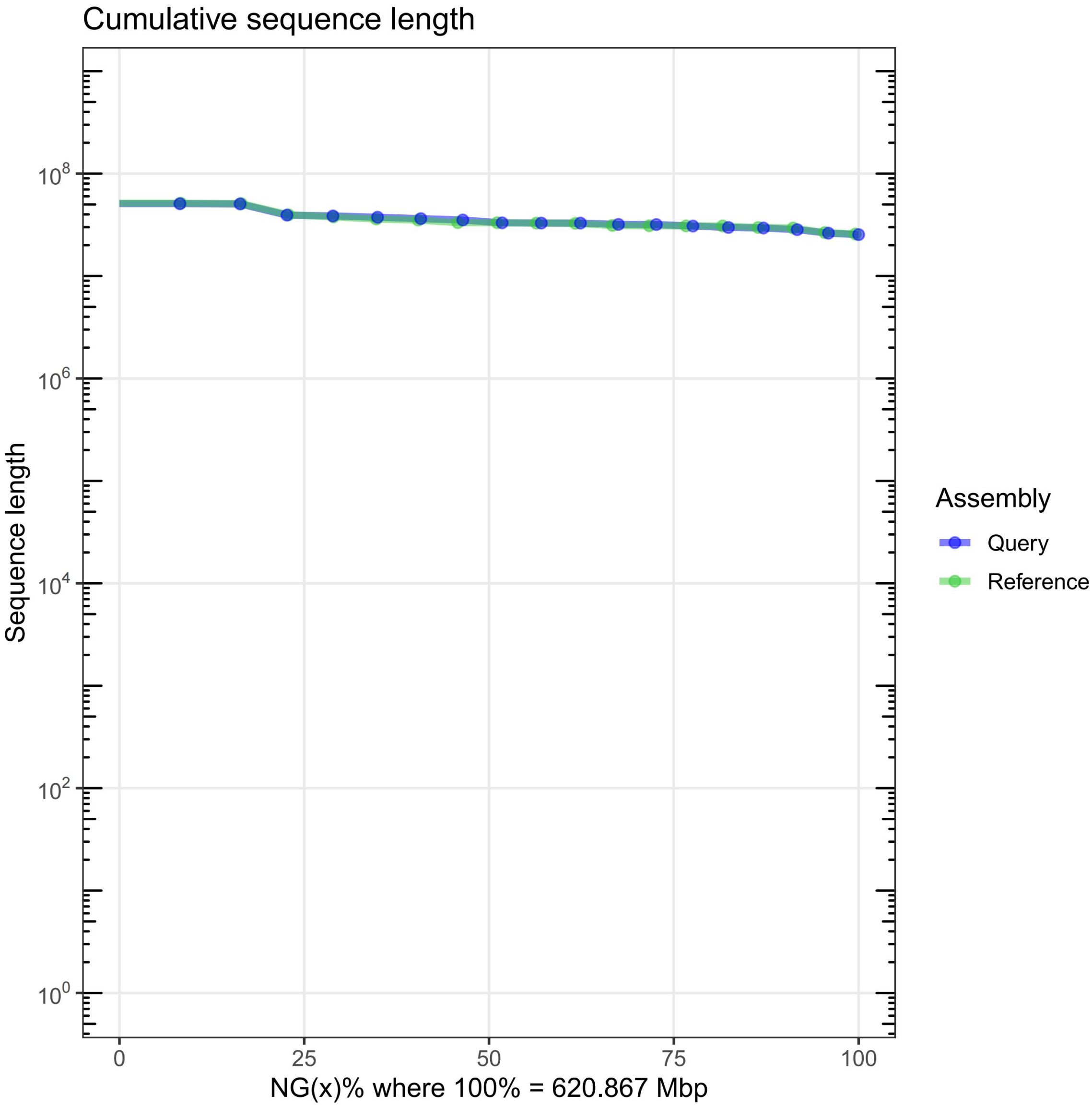

Supplementary Figure 1. Cumulative sequence length plot.

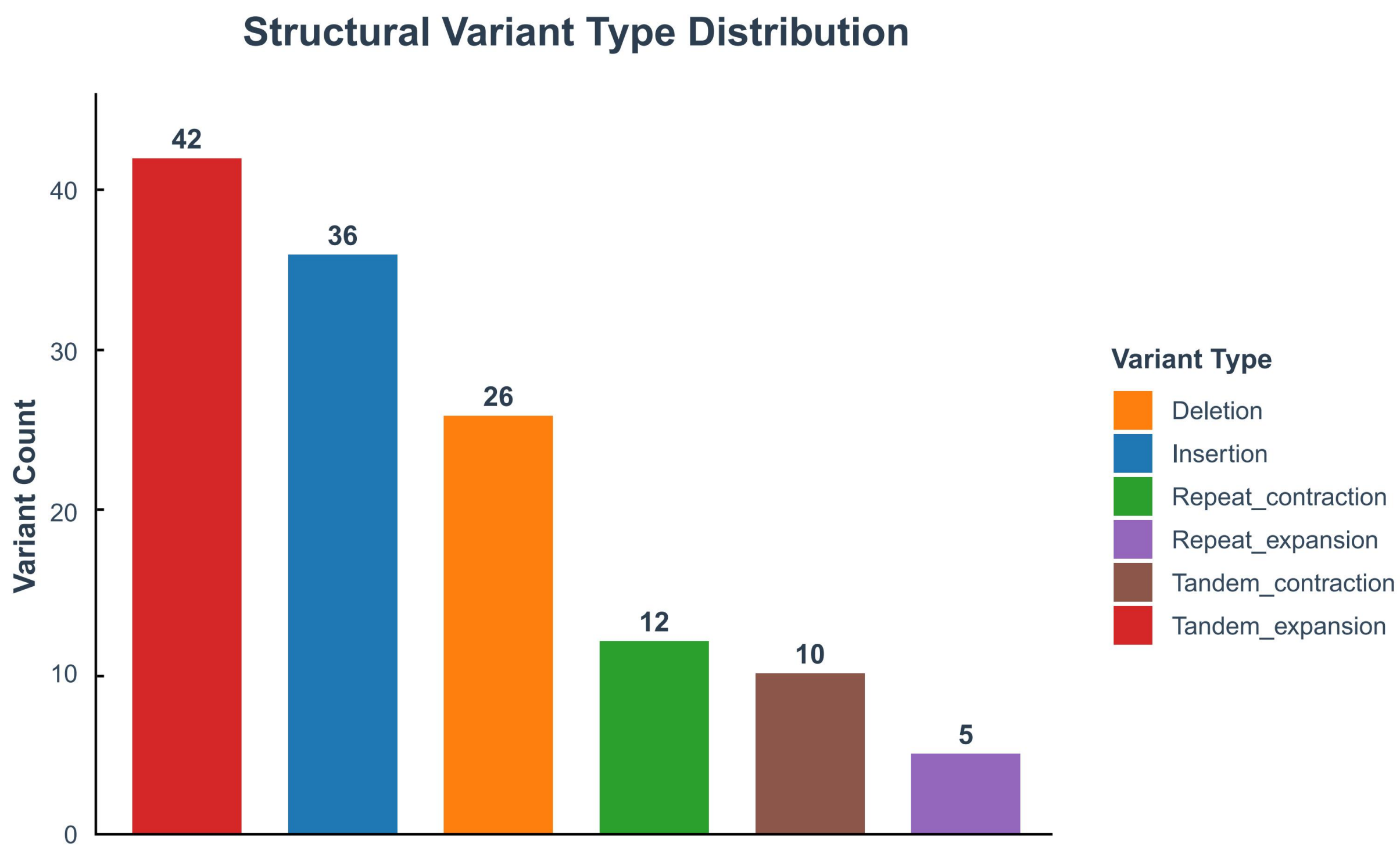

Supplementary Figure 2. Structural variation statistics plot.

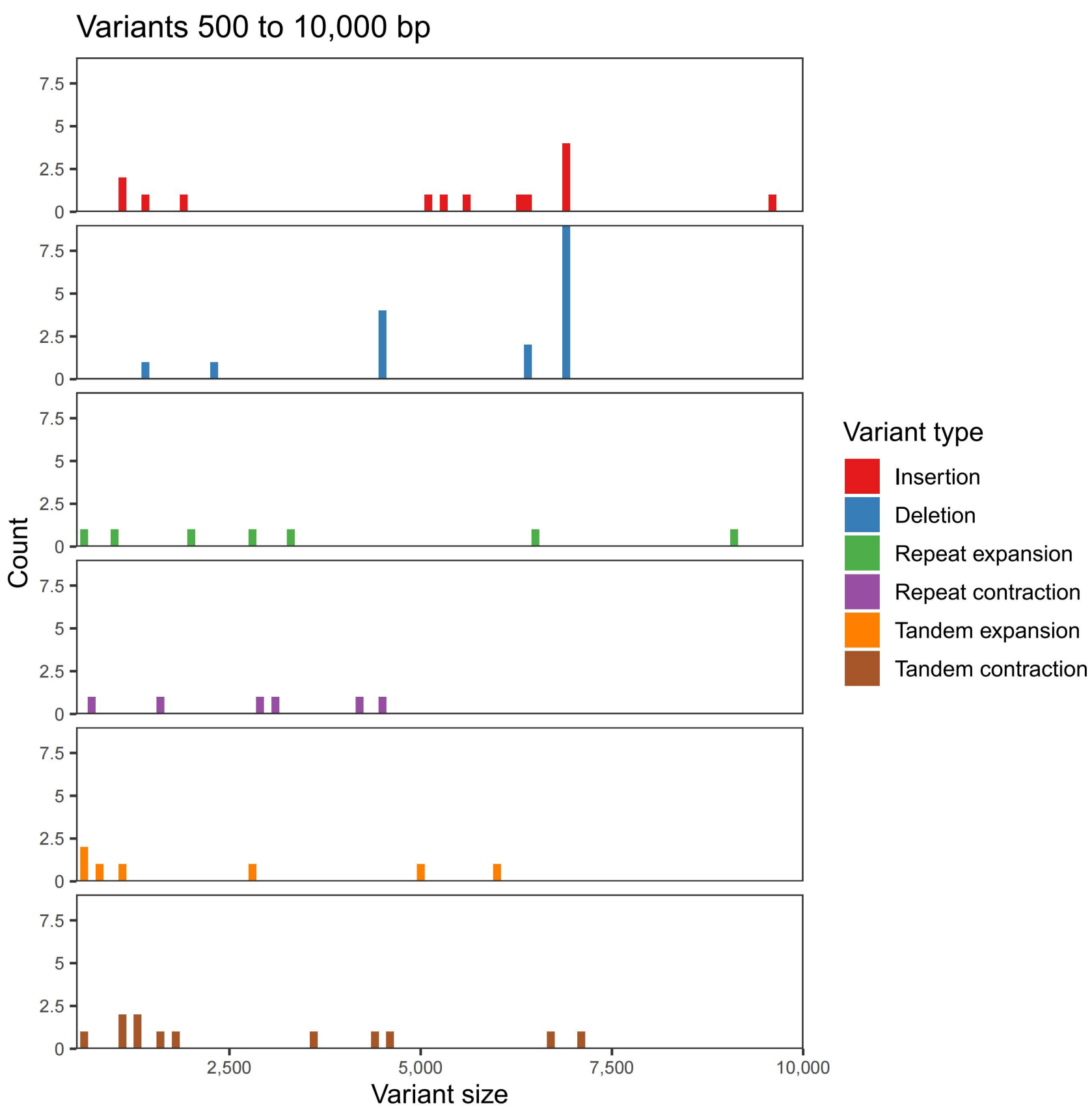

Supplementary Figure 3. Structural variant size distribution (500–10,000 bp).

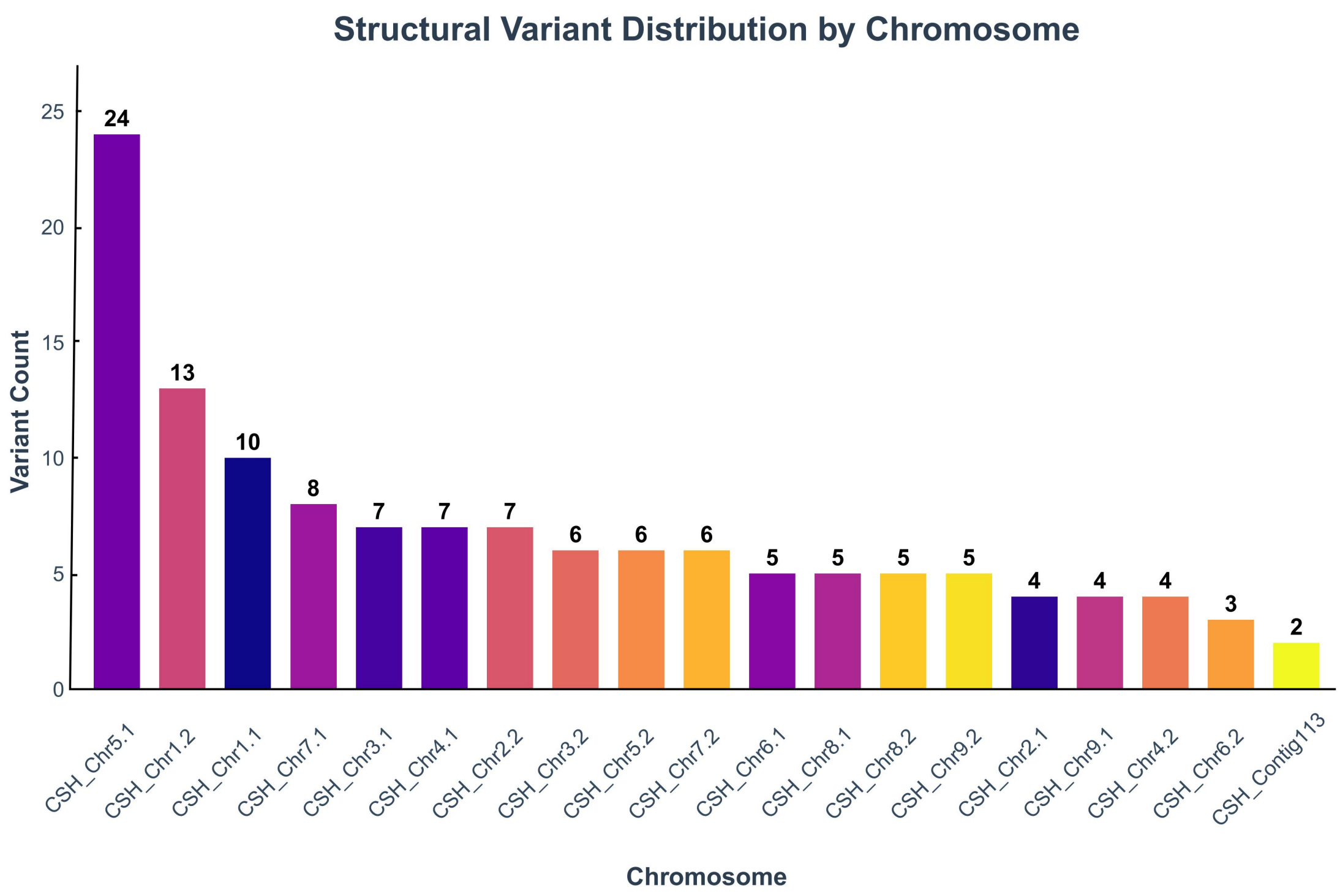

Supplementary Figure 4. Structural Variant Distribution by Chromosome.

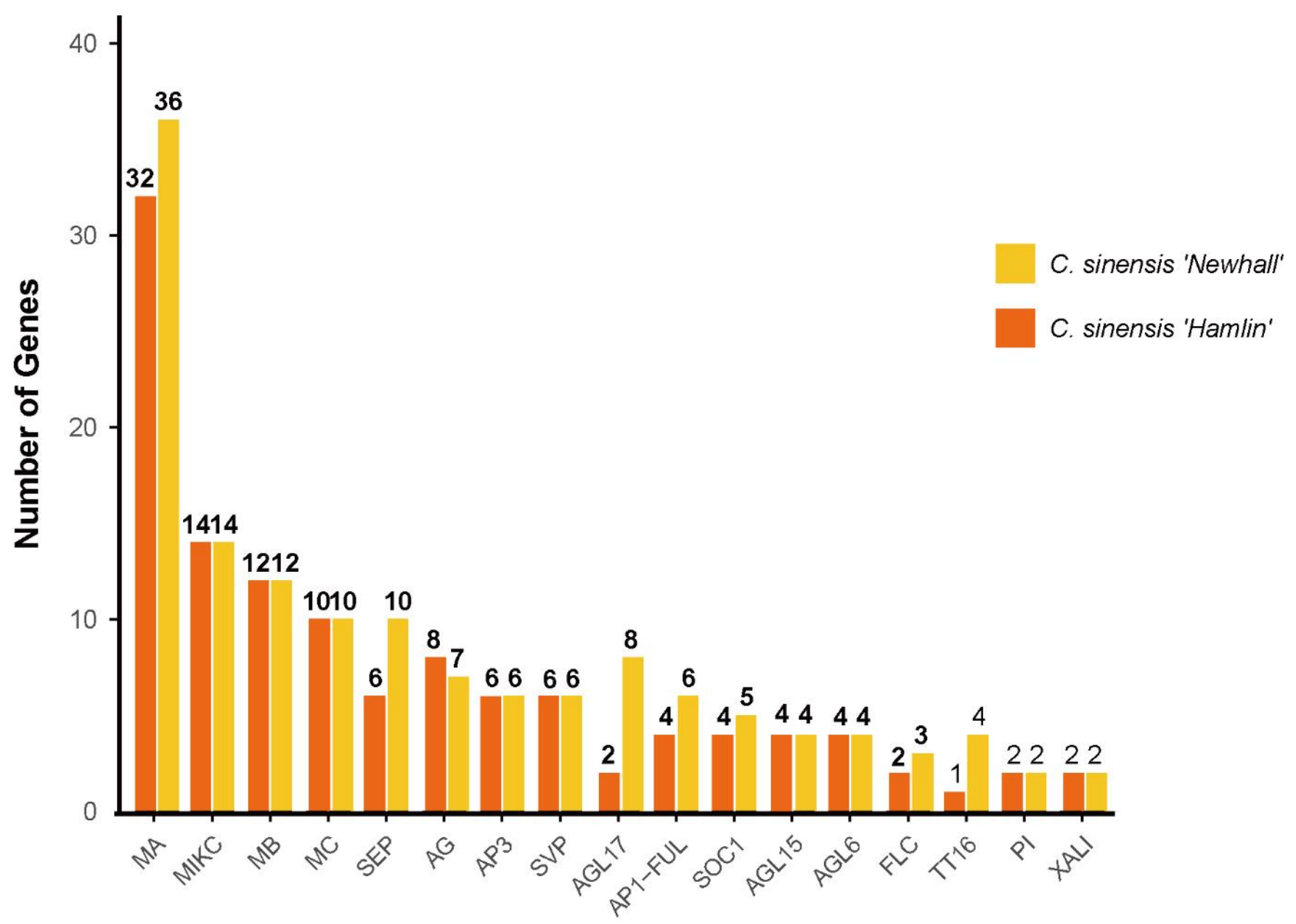

Supplementary Figure 5. Classification of MADS-box genes in Newhall navel oranges and Hamlin sweet oranges.

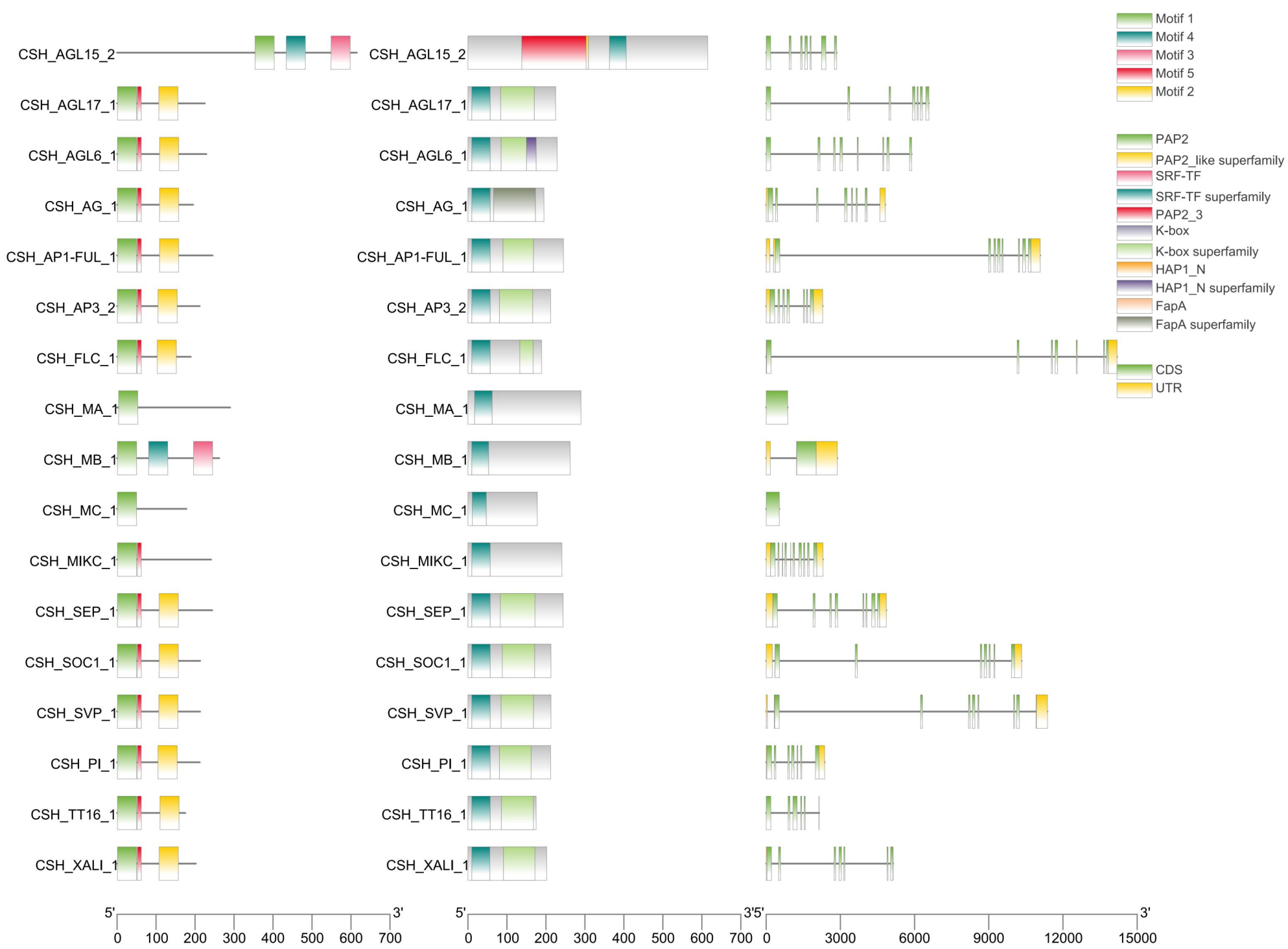

Supplementary Figure 6. Conserved motif, gene structure, and conserved domain analysis of MADS-box family genes in *Citrus sinensis* 'Hamlin'.

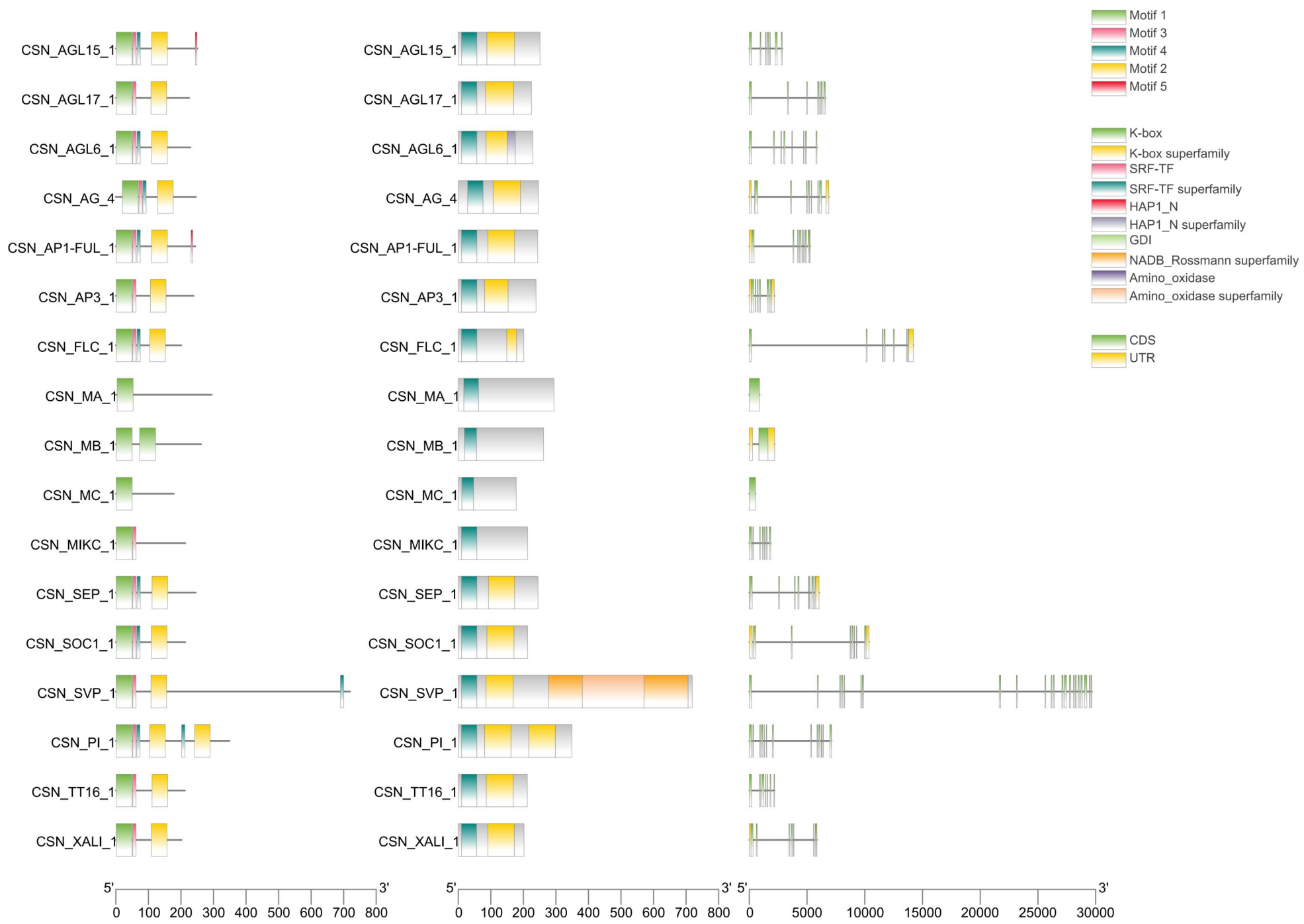

Supplementary Figure 7. Conserved motif, gene structure, and conserved domain analysis of MADS-box genes of *Citrus sinensis* 'Newhall'.

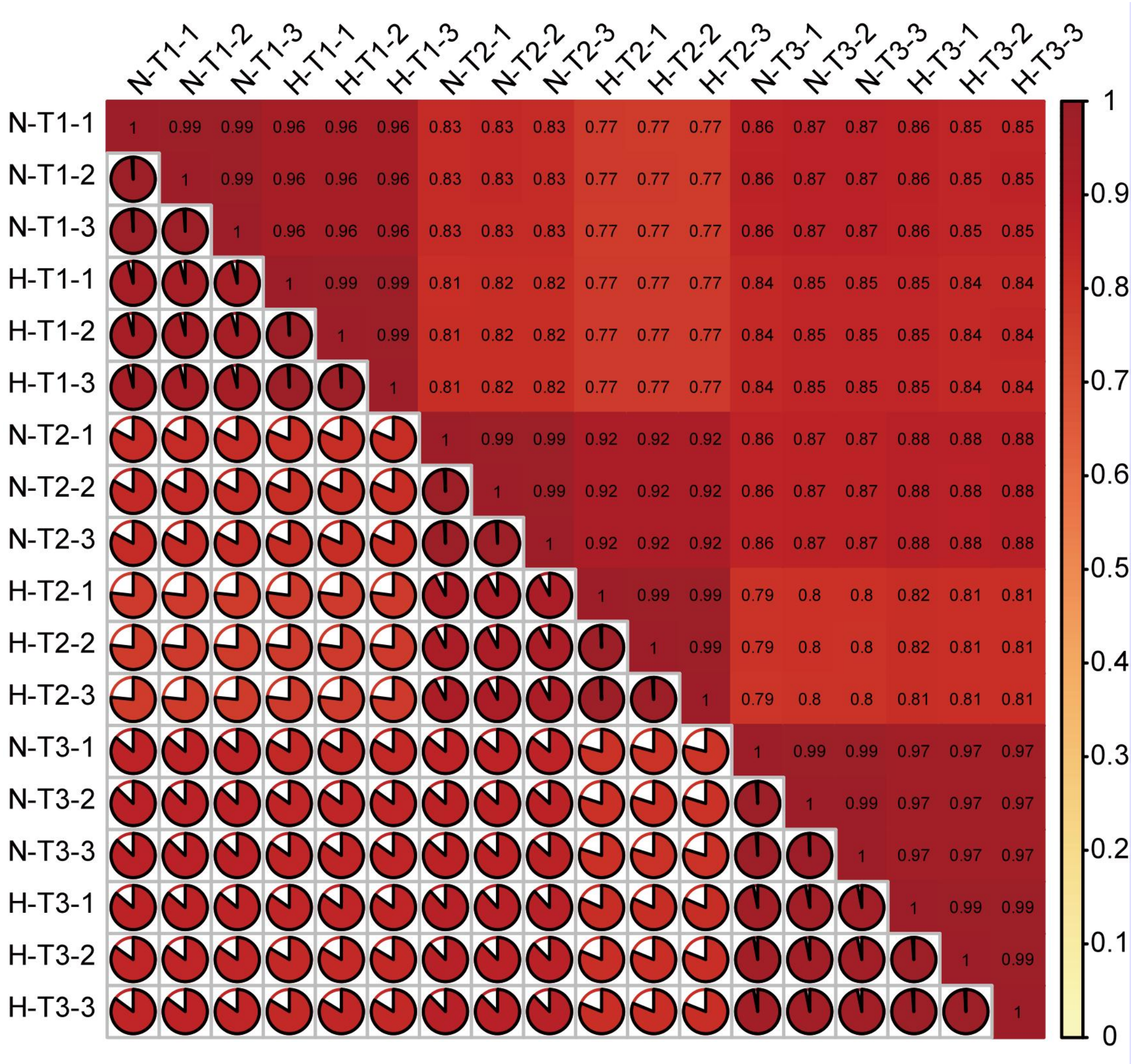

Supplementary Figure 8. Pearson correlation analysis between samples.The heatmap displays the pairwise correlation coefficients of gene expression levels among all samples. The upper triangle shows the correlation values, while the lower triangle presents scatter plots with fitted regression lines. High correlation coefficients (deep red color, >0.9) within biological replicates confirm the reliability and reproducibility of the RNA-seq data.

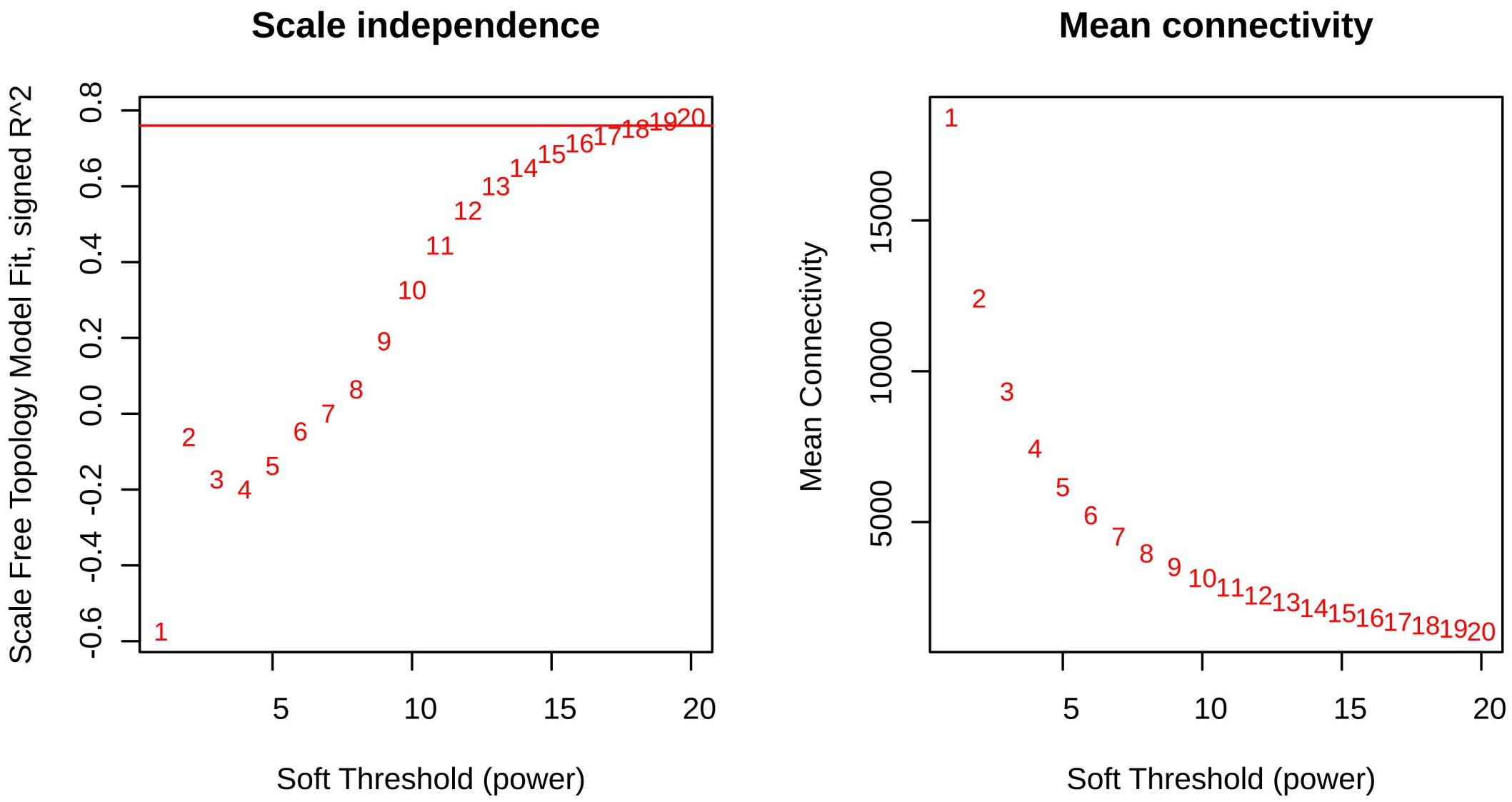

Supplementary Figure 9. Selection of soft-thresholding power for WGCNA analysis.

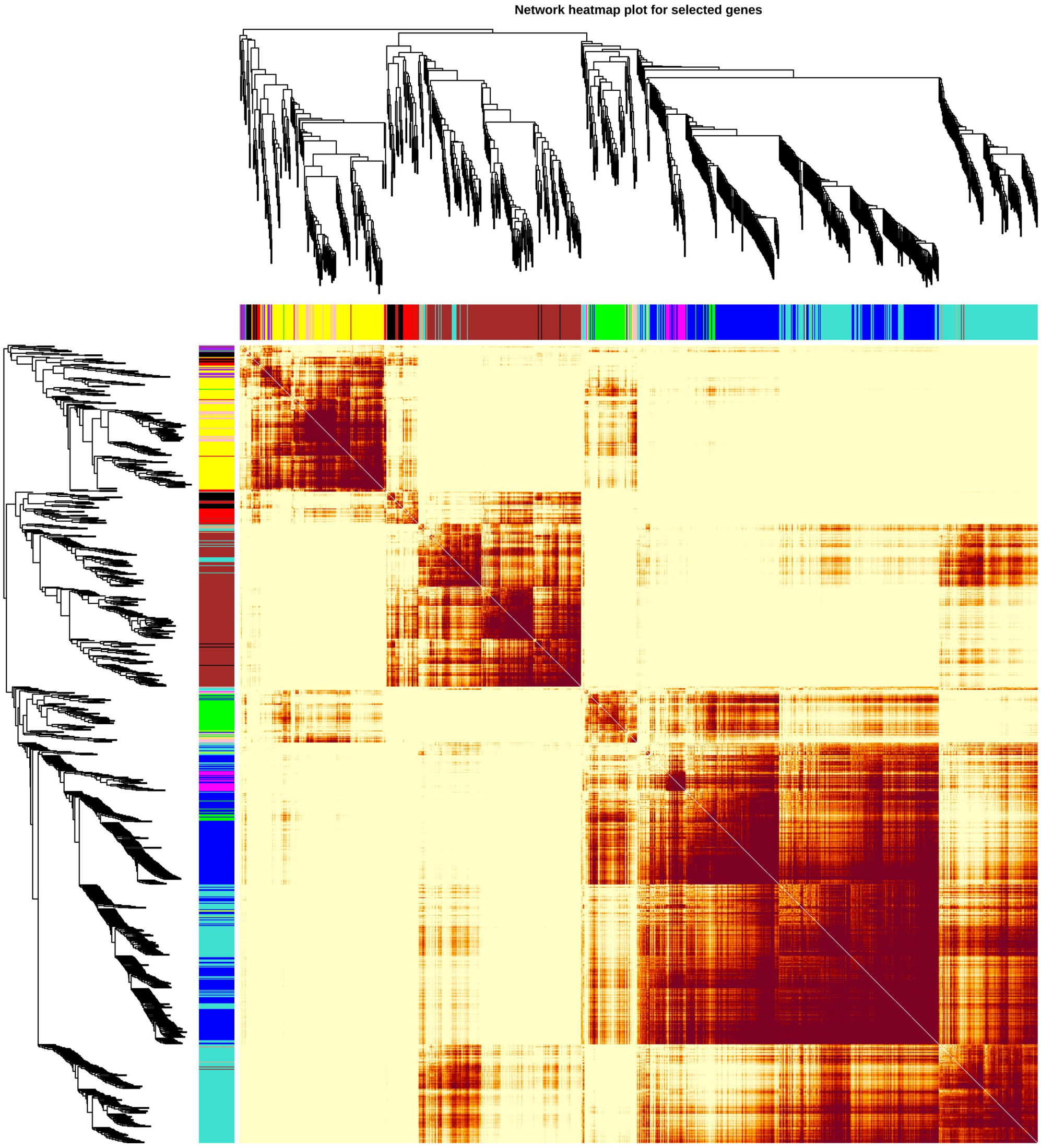

Supplementary Figure 10. Topological overlap matrix (TOM) heatmap of gene co- expression network. Rows and columns correspond to ordered genes sorted by hierarchical clustering dendrograms shown at the top and left margins, and the multicolored side strips denote distinct WGCNA gene modules. Gradient reddish-brown color intensity represents the magnitude of pairwise topological overlap between genes, with darker color indicating stronger co-expression connectivity; obviously concentrated dark-red blocks visually demonstrate tight intra-module gene correlation and weak inter-module linkage in the constructed co-expression network.

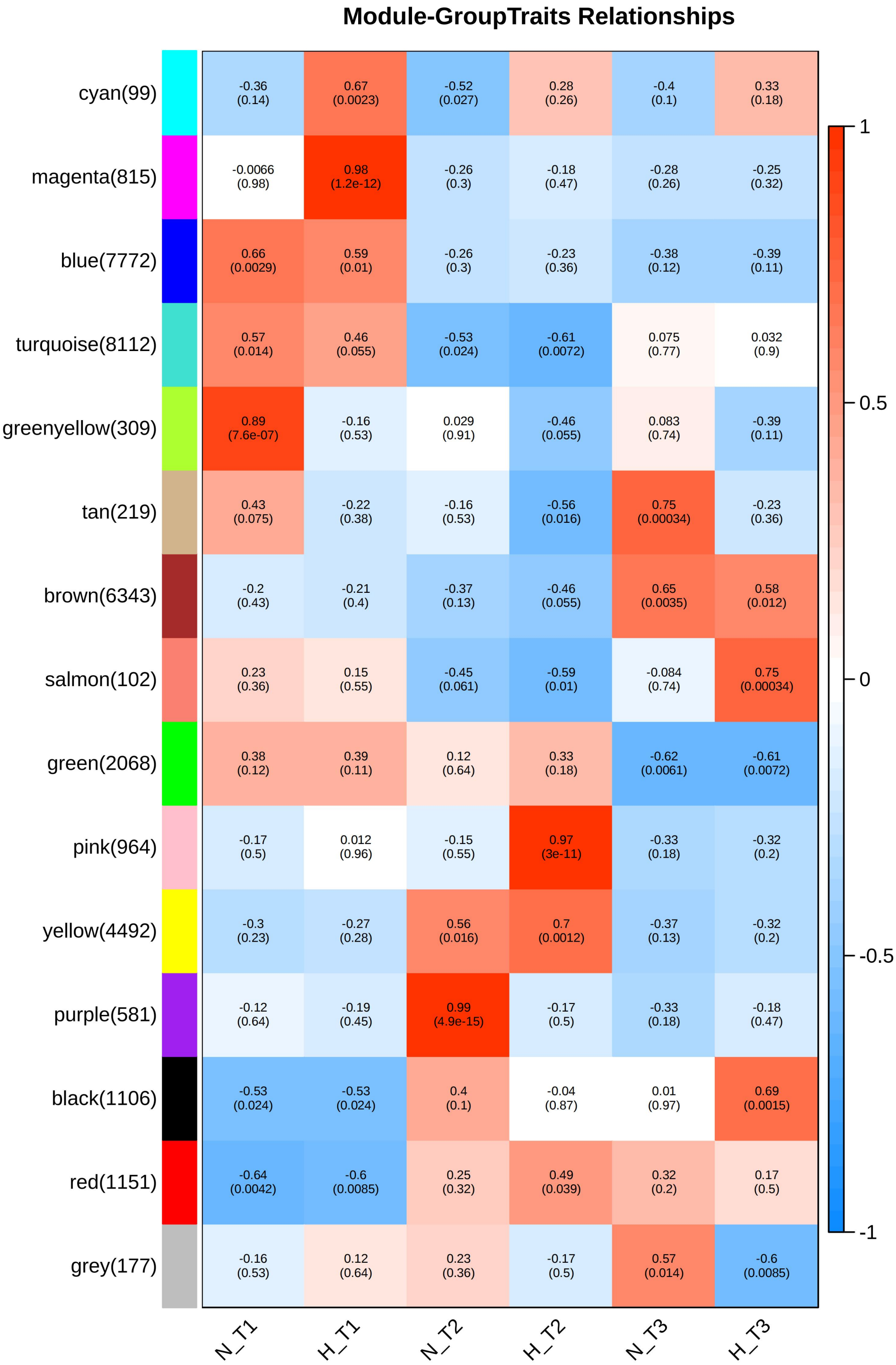

Supplementary Figure 11. Correlation heatmap between gene modules and sample group traits.

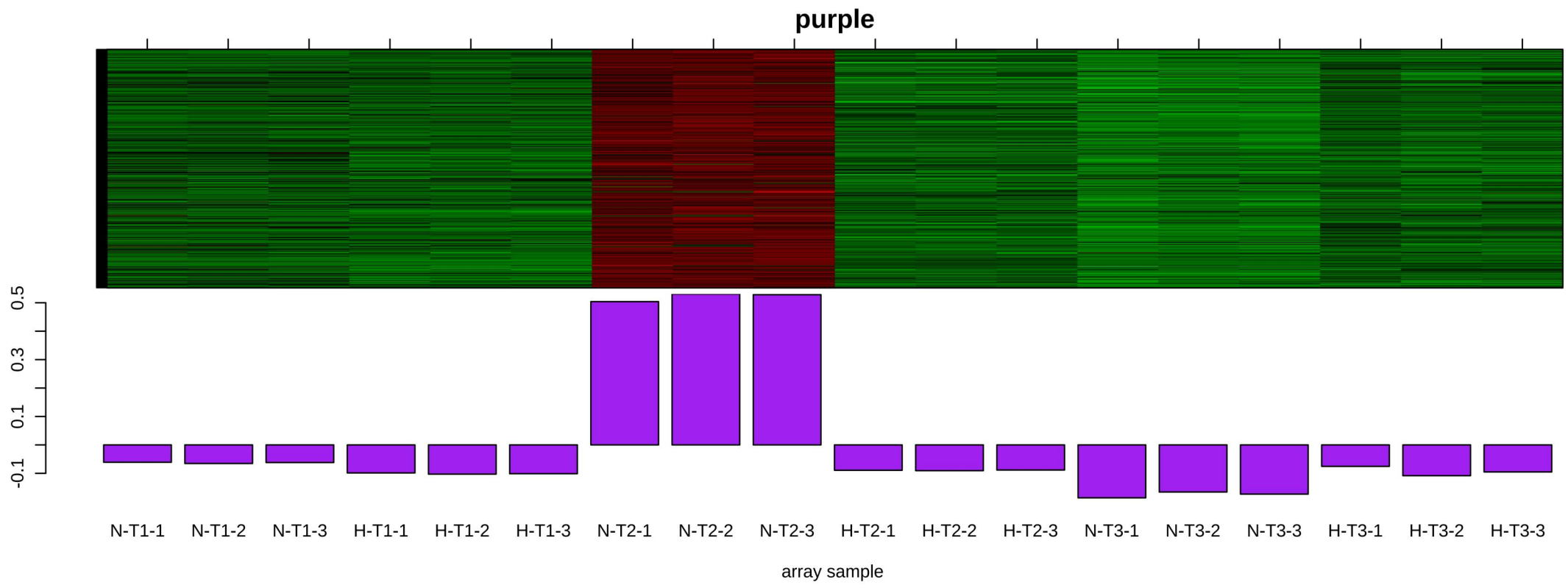

Supplementary Figure 12. Expression heatmap and module eigengene (ME) expression pattern of the purple module. Top panel: heatmap of normalized transcript abundance for all purple-module genes (red = high expression, green = low expression). Bottom panel: bar chart of module eigengene values across samples; ME value is markedly upregulated exclusively in N-T2 replicates, consistent with the gene upregulation observed in the heatmap of N-T2 samples.

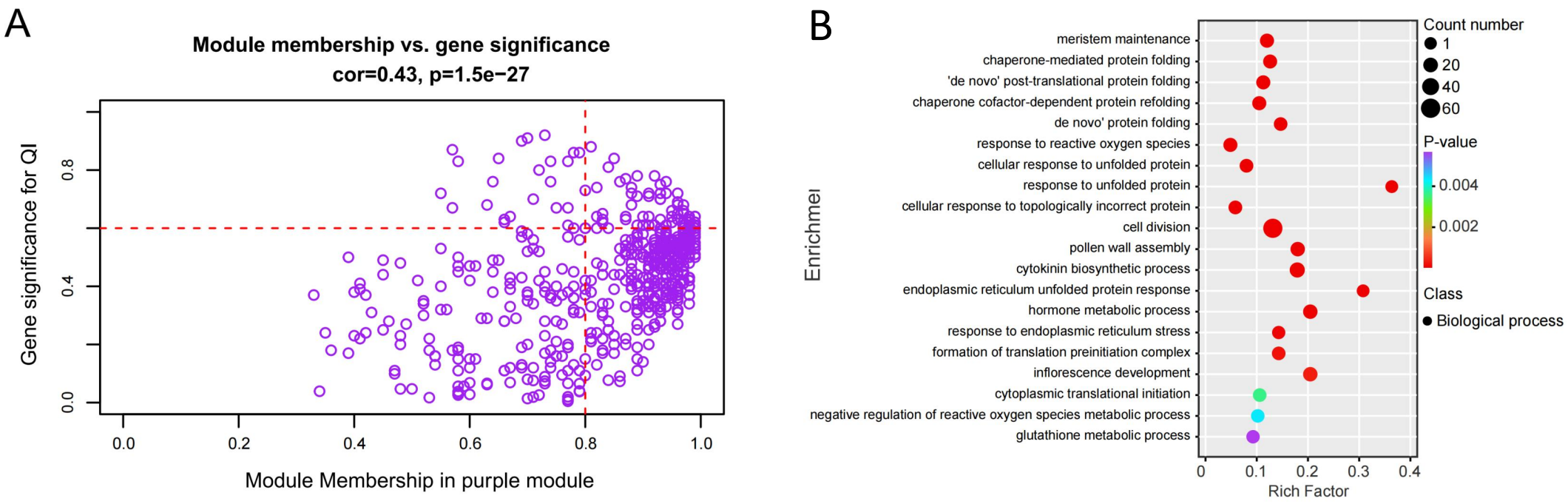

Supplementary Figure 14. Correlation between module membership and gene significance and functional enrichment analysis of hub modules. (A) Scatter plots showing the correlation between module membership (MM, x-axis) and gene significance (GS, y-axis) in purple module. Pearson correlation coefficient and corresponding P-value are annotated at the top of each panel; vertical and horizontal dashed red lines represent the cutoffs of MM = 0.8 and GS = 0.6 for screening hub genes, respectively. (B) GO biological process enrichment bubble plots of candidate hub genes derived from purple module. The x-axis denotes Rich factor, dot size corresponds to enriched gene count, and gradient color represents the significance of enrichment P-value.

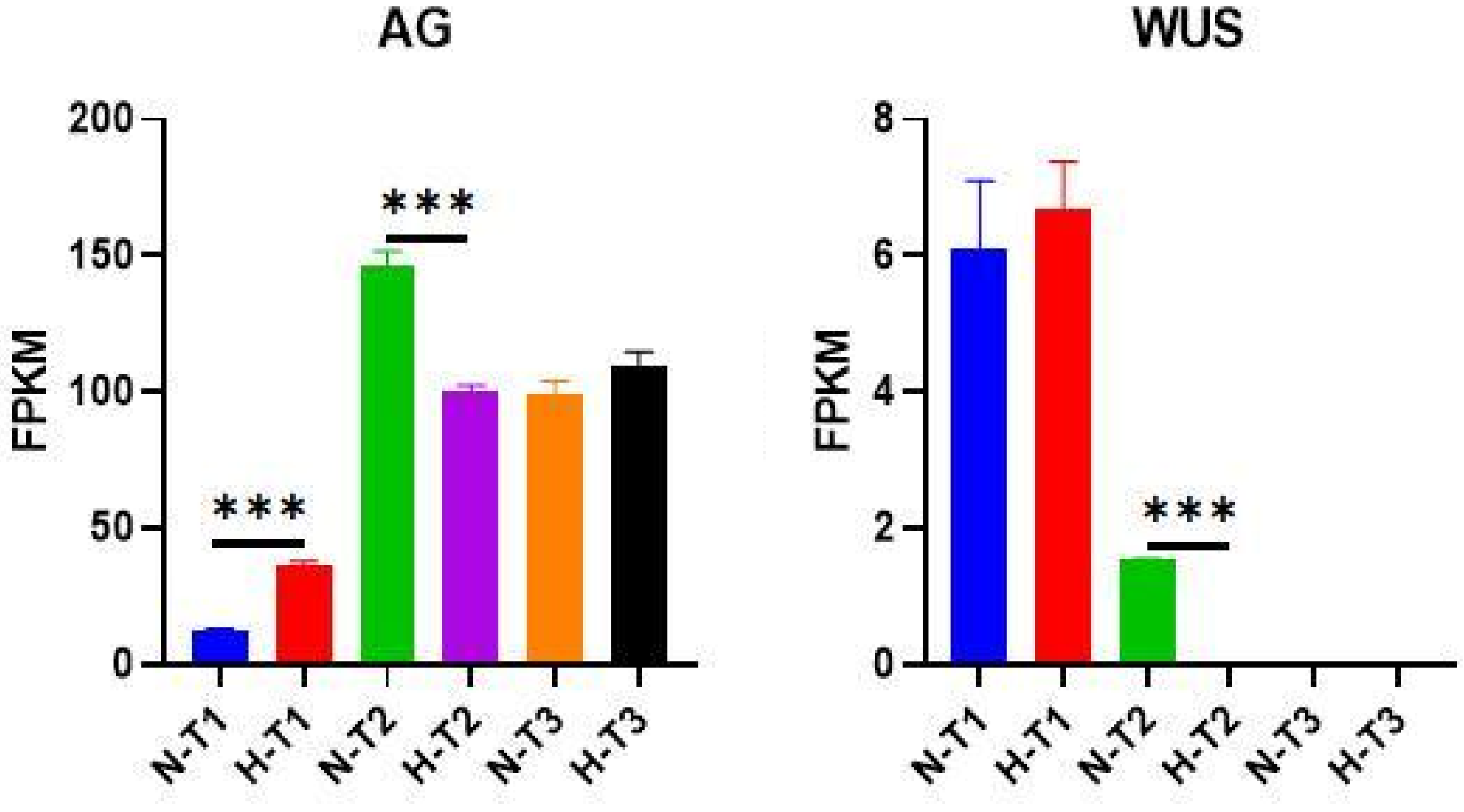

Supplementary Figure 15. Expression analysis of AG and WUS genes

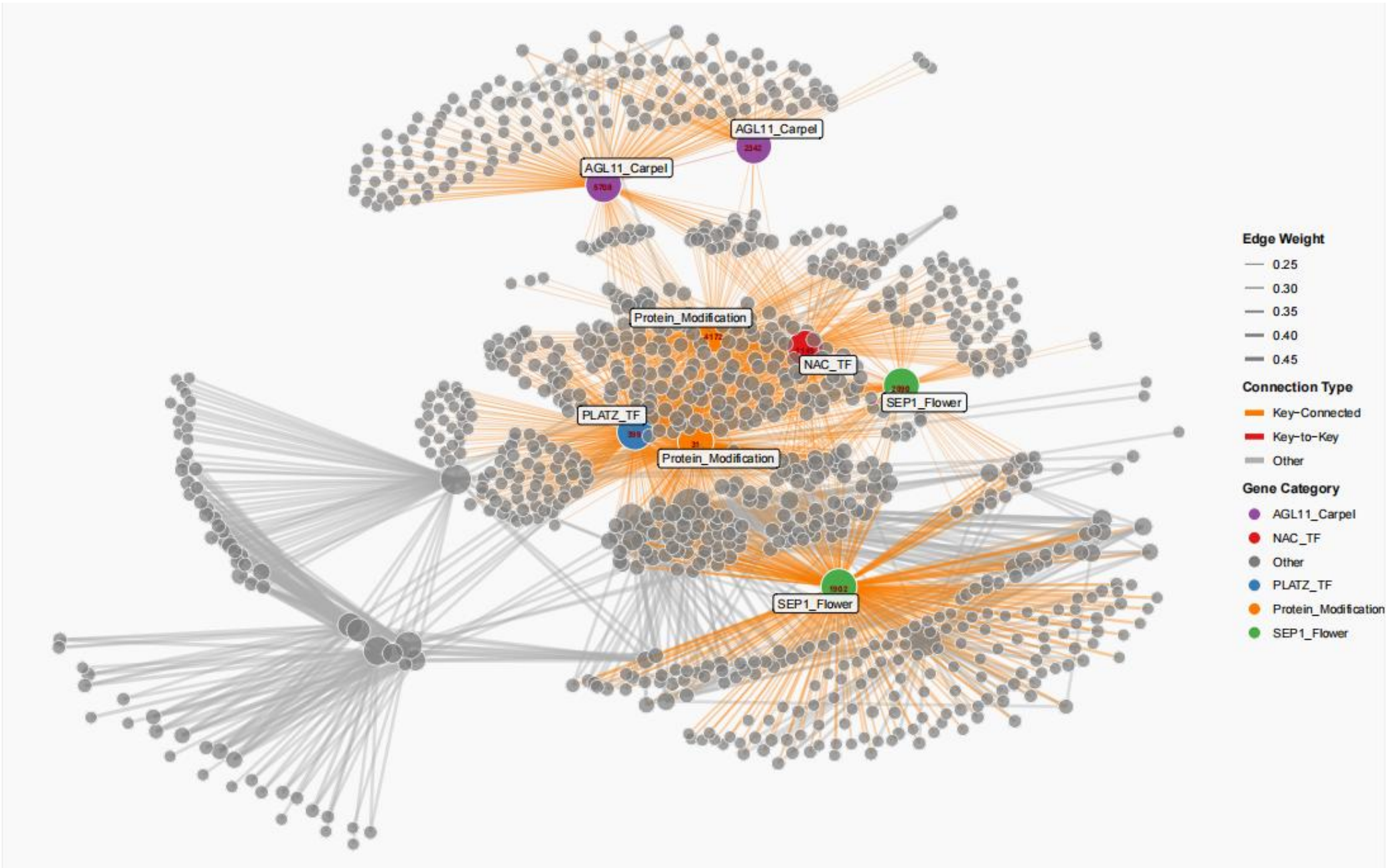

Supplementary Figure 16. the hub-gene interaction network.
