# Supplementary material for: Chromosome-level genome assembly of Hamlin sweet orange and transcriptomic insights into MADS-box gene regulation of secondary fruit development
Source: Front Plant Sci. 2026 Sep 8;17:1925055. doi: 10.3389/fpls.2026.1925055 (PMC13593871; doi:10.3389/fpls.2026.1925055)
Supplement: Supplementary file 2 [file DataSheet2.pdf]

Supplementary Table **1: Primer design information for QRCR-related genes**

ID	Sequence	Usage
evm.TU.Chr1.1997-qF	TGGAGAGGTTTCAGCAAGCA	qRT-PCR
evm.TU.Chr1.1997-qR	TCCGGGTGAGAGTAAGCAGT	
evm.TU.Chr8.1346-qF	GAGGAGGGCATGTGTACGAG	
evm.TU.Chr8.1346-qR	ACAAGTTGAAGAGGGGGCTG	
evm.TU.Chr6.170-qF	TAGCAGTGTTGTTGGCGGAT	
evm.TU.Chr6.170-qR	TTGCCATCCGCAGAGTTCTT	
evm.TU.Chr1.1118-qF	CTTACCAAATGGGAGGGGGC	
evm.TU.Chr1.1118-qR	TGAAGGTGACGAAGCATCCA	
evm.TU.Chr1.4019-qF	GGCCATTCAAGACCTGTGGA	
evm.TU.Chr1.4019-qR	TTTGTAGAGCAGGTTCCGGG	
evm.TU.Chr2.1893-qF	AGCAATATCACCGGCAGTCA	
evm.TU.Chr2.1893-qR	TCCCCATTTAGGTGCCTGAG	
evm.TU.Chr1.1117-qF	CCATTGAAACTCAGAGCACCT	
evm.TU.Chr1.1117-qR	CGGAGTGGTAAGGCAAGTGTA	
evm.TU.Chr1.1092-qF	GTGGTGTCTCCTGCACTTGA	
evm.TU.Chr1.1092-qR	CAGCAGGGGCCATATTTCCA	
evm.TU.chr1.1.3823-qF	CAGCAGGGGCCATATTTCCA	
evm.TU.chr1.1.3823-qR	CTTGCCAGCATGAGACCACT	
evm.TU.chr1.1.4125-qF	GCTTACGGGAGATGAAGCGA	
evm.TU.chr1.1.4125-qR	GTGATGGTGGTGGTACAGGG	
evm.TU.Chr1.2908-qF	TGGGACCAAGATTCGAGCAC	
evm.TU.Chr1.2908-qR	ACTTTTGCTGGCAATCCACG	
evm.TU.Chr1.2150-qF	GCAGTTCTTGCTGATGGGTG	
evm.TU.Chr1.2150-qR	CTGGCAAAAACGCCTTCCAC	
evm.TU.Chr1.3057-qF	GGCACTGACATCCCCAAGAA	
evm.TU.Chr1.3057-qR	TCACTTCACCTCCCTCGGAA	
evm.TU.Chr2.1619-qF	TGATGGACGAGCCTTCACAC	

evm.TU.Chr2.1619-qR	CACAGAGCCATTAGCTGCCT
evm.TU.Chr5.1540-qF	GAGCTTTTGGGTTTGCCACC
evm.TU.Chr5.1540-qR	CAATGTTCTGGTGCAAGGGC

Supplementary Table **2: Genome integrity assessment**

**Software**	**Results**
CEGMA	Prots：232，Completeness:93.55%
BWA	Mappingrate:97.8%，coverage:99.83%
Samtools	AllSNP:1.023903%;HomologySNP:1.023816%；HeterozygosisSNP:8.7e-05%

Supplementary Table **3: Length and number of clusters for each chromosome in the genome**

Sequences ID	Cluster Number	Sequences Length
Chr1.1	1	50,572,616
Chr2.1	1	37,511,817
Chr3.1	1	29,445,786
Chr4.1	2	36,346,236
Chr5.1	4	32,891,559
Chr6.1	3	31,905,808
Chr7.1	3	35,239,691
Chr8.1	2	28,360,140
Chr9.1	2	26,201,013
Chr1.2	3	50,784,892
Chr2.2	2	39,318,791
Chr3.2	1	38,616,373
Chr4.2	1	33,056,274
Chr5.2	2	32,875,402
Chr6.2	3	31,816,111
Chr7.2	2	30,762,641
Chr8.2	2	29,795,974
Chr9.2	2	25,366,080

Supplementary Table **4: Copy numbers of the MADS-box gene family in two sweet orange varieties**

MADS-box Gene	Copy number	
	CSH	CSN
AG	8	7
AGL15	4	4
AGL17	2	8
AGL6	4	4
AP1-FUL	4	6
AP3	6	6
FLC	2	3
MA	32	36
MB	12	12
MC	10	10
MIKC	14	14
PI	2	2
SEP	6	10
SOC1	4	5
SVP	6	6
TT16	1	4
XALI	2	2

Supplementary Table 5: **Physicochemical properties and subcellular localization prediction of MADS gene family proteins**

Sequence ID	Number of Amino Acid	Molecular Weight	Theoretical pI	Instability Index	Aliphatic Index	Grand Average of Hydropathicity	Subcellular localization
CSH_AG_1	194	22724.41	9.73	56.24	90	-0.745	Nucleus
CSH_AG_2	194	22724.41	9.73	56.24	90	-0.745	Nucleus
CSH_AG_3	257	29644.62	9.37	64.42	74.82	-0.852	Nucleus
CSH_AG_4	257	29644.62	9.37	64.42	74.82	-0.852	Nucleus
CSH_AG_5	245	28191.01	9.59	49.32	74.9	-0.774	Nucleus
CSH_AG_6	245	28191.01	9.59	49.32	74.9	-0.774	Nucleus
CSH_AG_7	226	25840.53	9.44	54.76	86.77	-0.577	Nucleus
CSH_AG_8	226	25840.53	9.44	54.76	86.77	-0.577	Nucleus
CSH_AGL15_1	241	27093.98	8.29	55.07	88.63	-0.576	Nucleus
CSH_AGL15_2	241	27093.98	8.29	55.07	88.63	-0.576	Nucleus
CSH_AGL15_3	253	28881.73	7.69	62.15	76.68	-0.796	Nucleus
CSH_AGL15_4	253	28881.73	7.69	62.15	76.68	-0.796	Nucleus
CSH_AGL17_1	224	25839.72	9.35	38.59	95.76	-0.617	Nucleus
CSH_AGL17_2	224	25839.72	9.35	38.59	95.76	-0.617	Nucleus
CSH_AGL6_1	228	26103.93	9.03	45.32	91.67	-0.509	Nucleus
CSH_AGL6_2	228	26103.93	9.03	45.32	91.67	-0.509	Nucleus
CSH_AGL6_3	244	27757.55	9.08	46.23	83.57	-0.67	Nucleus
CSH_AGL6_4	244	27757.55	9.08	46.23	83.57	-0.67	Nucleus
CSH_AP1-FUL_1	244	28401.42	9.06	50.28	82.75	-0.827	Nucleus
CSH_AP1-FUL_2	244	28401.42	9.06	50.28	82.75	-0.827	Nucleus
CSH_AP1-FUL_3	243	28096.2	8.9	58.6	86.75	-0.707	Nucleus
CSH_AP1-FUL_4	243	28096.2	8.9	58.6	86.75	-0.707	Nucleus
CSH_AP3_1	211	24364.9	8.59	49.12	85.02	-0.669	Nucleus
CSH_AP3_2	211	24364.9	8.59	49.12	85.02	-0.669	Nucleus
CSH_AP3_3	221	25873.6	9.51	55.45	88.19	-0.824	Nucleus
CSH_AP3_4	223	26118.9	9.51	55.04	87.4	-0.824	Nucleus
CSH_AP3_5	223	25761.15	9.39	44.23	69.1	-0.856	Nucleus
CSH_AP3_6	223	25761.15	9.39	44.23	69.1	-0.856	Nucleus
CSH_FLC_1	188	21016.34	9.43	43.75	98.67	-0.339	Nucleus
CSH_FLC_2	188	21016.34	9.43	43.75	98.67	-0.339	Nucleus
CSH_MA_1	289	32301.35	5.94	39.24	79.34	-0.453	Nucleus
CSH_MA_10	67	7695.14	10.61	51.98	81.49	-0.391	Nucleus
CSH_MA_11	237	27160.7	6.02	36.27	73.59	-0.808	Nucleus

CSH_MA_12	237	27160.7	6.02	36.27	73.59	-0.808	Nucleus
CSH_MA_13	216	24861.6	6.86	73.19	75.83	-0.628	Nucleus
CSH_MA_14	216	24861.6	6.86	73.19	75.83	-0.628	Nucleus
CSH_MA_15	168	18937.58	6.85	62.91	70.3	-0.654	Nucleus
CSH_MA_16	168	18937.58	6.85	62.91	70.3	-0.654	Nucleus
CSH_MA_17	219	24849.17	5.49	37.42	83.29	-0.631	Nucleus
CSH_MA_18	219	24849.17	5.49	37.42	83.29	-0.631	Nucleus
CSH_MA_19	226	25542.17	9.13	49.65	81.59	-0.617	Nucleus
CSH_MA_2	289	32301.35	5.94	39.24	79.34	-0.453	Nucleus
CSH_MA_20	226	25542.17	9.13	49.65	81.59	-0.617	Nucleus
CSH_MA_21	227	25614.11	9.34	50.63	78.24	-0.673	Nucleus
CSH_MA_22	79	8872.27	9.57	38.01	82.66	-0.347	Nucleus
CSH_MA_23	79	8872.27	9.57	38.01	82.66	-0.347	Nucleus
CSH_MA_24	152	17306.49	5.71	59.56	84.67	-0.53	Nucleus
CSH_MA_25	152	17306.49	5.71	59.56	84.67	-0.53	Nucleus
CSH_MA_26	210	24217.64	9.15	53.34	79	-0.745	Nucleus
CSH_MA_27	210	24217.64	9.15	53.34	79	-0.745	Nucleus
CSH_MA_28	170	19570.51	9.6	37.78	70.59	-0.819	Nucleus
CSH_MA_29	187	21471.43	9.35	47.07	74.65	-0.816	Nucleus
CSH_MA_3	405	46986.39	8.94	45.4	90	-0.282	Nucleus
CSH_MA_30	191	21577.45	6.32	46.44	69.01	-0.764	Nucleus
CSH_MA_31	191	21577.45	6.32	46.44	69.01	-0.764	Nucleus
CSH_MA_32	67	7256.56	8.12	34.37	132.54	0.331	Nucleus
CSH_MA_4	405	46986.39	8.94	45.4	90	-0.282	Nucleus
CSH_MA_5	170	18828.65	9.06	51.52	84.88	-0.338	Nucleus
CSH_MA_6	170	18828.65	9.06	51.52	84.88	-0.338	Nucleus
CSH_MA_7	170	18858.68	9.06	55.21	84.88	-0.34	Nucleus
CSH_MA_8	170	18858.68	9.06	55.21	84.88	-0.34	Nucleus
CSH_MA_9	67	7695.14	10.61	51.98	81.49	-0.391	Nucleus
CSH_MB_1	261	29056.13	9.01	35.32	72.95	-0.666	Nucleus
CSH_MB_10	286	32233.62	5.37	46.98	70.87	-0.644	Nucleus
CSH_MB_11	275	31184.61	4.46	36	71.53	-0.579	Nucleus
CSH_MB_12	275	31184.61	4.46	36	71.53	-0.579	Nucleus
CSH_MB_2	614	69343.82	8.51	39.12	86.53	-0.159	Nucleus
CSH_MB_3	261	29153.2	8.7	39.23	70.27	-0.668	Nucleus
CSH_MB_4	261	29153.2	8.7	39.23	70.27	-0.668	Nucleus
CSH_MB_5	271	31531.63	8.89	34.31	72.32	-0.469	Nucleus
CSH_MB_6	271	31531.63	8.89	34.31	72.32	-0.469	Nucleus
CSH_MB_7	315	36320.8	9.45	22.42	68.98	-0.539	Nucleus
CSH_MB_8	315	36320.8	9.45	22.42	68.98	-0.539	Nucleus
CSH_MB_9	286	32233.62	5.37	46.98	70.87	-0.644	Nucleus
CSH_MC_1	177	20376.84	9.76	45.09	74.97	-0.676	Nucleus
CSH_MC_10	233	27060.41	9.5	58.56	68.63	-0.674	Nucleus
CSH_MC_2	177	20376.84	9.76	45.09	74.97	-0.676	Nucleus

CSH_MC_3	446	48440.38	6.88	52.2	71.12	-0.646	Nucleus
CSH_MC_4	446	48440.38	6.88	52.2	71.12	-0.646	Nucleus
CSH_MC_5	237	27199.28	9.3	57.18	69.96	-0.691	Chloroplast
CSH_MC_6	237	27199.28	9.3	57.18	69.96	-0.691	Nucleus
CSH_MC_7	237	27168.25	9.37	53.19	68.69	-0.706	Nucleus
CSH_MC_8	237	27168.25	9.37	53.19	68.69	-0.706	Nucleus
CSH_MC_9	233	27060.41	9.5	58.56	68.63	-0.674	Chloroplast
CSH_MIKC_1	240	28037.13	7.64	63.79	78.42	-0.753	Nucleus
CSH_MIKC_10	360	40519.61	6.12	48.18	76.67	-0.614	Nucleus
CSH_MIKC_11	349	39372.52	6.76	50.68	78.19	-0.617	Nucleus
CSH_MIKC_12	349	39372.52	6.76	50.68	78.19	-0.617	Nucleus
CSH_MIKC_13	507	57269.47	6.15	42.48	67.53	-0.825	Nucleus
CSH_MIKC_14	507	57269.47	6.15	42.48	67.53	-0.825	Nucleus
CSH_MIKC_2	240	28037.13	7.64	63.79	78.42	-0.753	Nucleus
CSH_MIKC_3	345	39236.25	7.14	61.38	73.3	-0.598	Nucleus
CSH_MIKC_4	345	39236.25	7.14	61.38	73.3	-0.598	Nucleus
CSH_MIKC_5	355	40810.86	5.52	56.83	79.32	-0.676	Nucleus
CSH_MIKC_6	355	40810.86	5.52	56.83	79.32	-0.676	Nucleus
CSH_MIKC_7	183	21405.18	10.53	55.54	83.11	-0.993	Nucleus
CSH_MIKC_8	183	21405.18	10.53	55.54	83.11	-0.993	Nucleus
CSH_MIKC_9	360	40519.61	6.12	48.18	76.67	-0.614	Nucleus
CSH_PI_1	211	24829.18	8.42	66.87	82.27	-0.95	Nucleus
CSH_PI_2	211	24829.18	8.42	66.87	82.27	-0.95	Nucleus
CSH_SEP_1	243	27954.86	9.01	41.82	82.67	-0.686	Nucleus
CSH_SEP_2	243	27954.86	9.01	41.82	82.67	-0.686	Nucleus
CSH_SEP_3	265	30215.63	8.9	51.53	86.49	-0.482	Nucleus
CSH_SEP_4	265	30215.63	8.9	51.53	86.49	-0.482	Nucleus
CSH_SEP_5	245	28035.67	7.8	53.01	81.27	-0.727	Nucleus
CSH_SEP_6	245	28035.67	7.8	53.01	81.27	-0.727	Nucleus
CSH_SOC1_1	212	24892.42	8.53	56.15	76.84	-0.847	Nucleus
CSH_SOC1_2	212	24892.42	8.53	56.15	76.84	-0.847	Nucleus
CSH_SOC1_3	202	23517.1	9.11	53.72	83.02	-0.772	Nucleus
CSH_SOC1_4	202	23517.1	9.11	53.72	83.02	-0.772	Nucleus
CSH_SVP_1	212	24317.51	8.52	58.57	79.58	-0.733	Nucleus
CSH_SVP_2	212	24317.51	8.52	58.57	79.58	-0.733	Nucleus
CSH_SVP_3	227	25359.96	8.6	59.8	88.9	-0.527	Nucleus
CSH_SVP_4	227	25359.96	8.6	59.8	88.9	-0.527	Nucleus
CSH_SVP_5	217	24674.98	6.18	57.03	87.19	-0.693	Nucleus
CSH_SVP_6	217	24674.98	6.18	57.03	87.19	-0.693	Nucleus
CSH_TT16_1	174	20054.03	8.68	62.01	93.56	-0.58	Nucleus
CSH_XALI_1	201	22901.67	7.68	55.6	91.74	-0.292	Nucleus
CSH_XALI_2	201	22901.67	7.68	55.6	91.74	-0.292	Nucleus
CSN_AG_1	184	21423.87	9.93	43.91	97.5	-0.692	Nucleus
CSN_AG_2	267	30623.7	9.28	61.26	78.99	-0.772	Nucleus

CSN_AG_3	267	30667.71	9.18	61.26	78.61	-0.792	Nucleus
CSN_AG_4	245	28191.01	9.59	49.32	74.9	-0.774	Nucleus
CSN_AG_5	252	29061.14	9.74	48.81	75.12	-0.775	Nucleus
CSN_AG_6	208	23727.28	9.6	49.93	86.78	-0.569	Nucleus
CSN_AG_7	310	34789.19	8.51	54.42	78.1	-0.512	Nucleus
CSN_AGL15_1	241	27093.98	8.29	55.07	88.63	-0.576	Nucleus
CSN_AGL15_2	241	27108.01	8.29	56.89	89.42	-0.566	Nucleus
CSN_AGL15_3	253	28853.71	7.68	60.84	76.68	-0.794	Nucleus
CSN_AGL15_4	253	28881.73	7.69	62.15	76.68	-0.796	Nucleus
CSN_AGL17_1	224	25839.72	9.35	38.59	95.76	-0.617	Nucleus
CSN_AGL17_2	224	25834.7	9.24	40.06	94.87	-0.619	Nucleus
CSN_AGL17_3	225	26140.79	7.79	49.42	96.62	-0.748	Nucleus
CSN_AGL17_4	225	26110.77	7.79	51.5	97.07	-0.737	Nucleus
CSN_AGL17_5	259	29387.47	9.08	48.55	88.46	-0.594	Nucleus
CSN_AGL17_6	259	29387.47	9.08	48.55	88.46	-0.594	Nucleus
CSN_AGL17_7	322	36817.3	9.69	49.68	84.47	-0.689	Nucleus
CSN_AGL17_8	271	31141.61	9.57	41.93	88.86	-0.768	Nucleus
CSN_AGL6_1	228	26118.01	9.18	45.16	91.67	-0.511	Nucleus
CSN_AGL6_2	201	23332.93	9.22	45.95	93.73	-0.568	Chloroplast
CSN_AGL6_3	244	27757.55	9.08	46.23	83.57	-0.67	Nucleus
CSN_AGL6_4	244	27757.55	9.08	46.23	83.57	-0.67	Nucleus
CSN_AP1-FUL_1	243	28404.38	9.16	55.2	81.81	-0.799	Chloroplast
CSN_AP1-FUL_2	243	28359.38	9.14	55.25	81.81	-0.783	Nucleus
CSN_AP1-FUL_3	244	28401.42	9.06	50.28	82.75	-0.827	Chloroplast
CSN_AP1-FUL_4	244	28401.42	9.06	50.28	82.75	-0.827	Nucleus
CSN_AP1-FUL_5	243	28039.14	8.9	57.77	86.75	-0.694	Chloroplast
CSN_AP1-FUL_6	243	28096.2	8.9	58.6	86.75	-0.707	Nucleus
CSN_AP3_1	238	27357.43	8.15	50.63	92.14	-0.445	Nucleus
CSN_AP3_2	238	27394.51	8.45	50.11	90.5	-0.461	Chloroplast
CSN_AP3_3	221	25874.64	9.66	52.63	88.64	-0.813	Nucleus
CSN_AP3_4	221	25873.6	9.51	55.45	88.19	-0.824	Nucleus
CSN_AP3_5	223	25809.2	9.39	44.23	67.8	-0.862	Mitochondrion
CSN_AP3_6	223	25761.15	9.39	44.23	69.1	-0.856	Nucleus
CSN_FLC_1	200	22395.88	9.4	41.16	98.6	-0.308	Nucleus
CSN_FLC_2	204	22812.31	9.13	37.16	99.07	-0.292	Nucleus
CSN_FLC_3	188	21016.34	9.43	43.75	98.67	-0.339	Cytoplasm
CSN_MA_1	293	32564.62	5.78	37.8	78.94	-0.416	Nucleus
CSN_MA_10	289	32301.35	5.94	39.24	79.34	-0.453	Nucleus
CSN_MA_11	149	16431.05	9.43	54.8	89.6	-0.242	Nucleus
CSN_MA_12	67	7709.21	10.66	54.23	81.49	-0.397	Chloroplast
CSN_MA_13	67	7709.21	10.66	54.23	81.49	-0.397	Nucleus
CSN_MA_14	67	7695.14	10.61	51.98	81.49	-0.391	Nucleus
CSN_MA_15	301	33731.37	8.76	32.02	78.31	-0.661	Nucleus
CSN_MA_16	237	27061.56	5.66	38.27	73.59	-0.784	Nucleus

CSN_MA_17	237	27160.7	6.02	36.27	73.59	-0.808	Chloroplast
CSN_MA_18	216	24861.6	6.86	73.19	75.83	-0.628	Nucleus
CSN_MA_19	215	24804.55	6.86	72.79	75.72	-0.639	Chloroplast
CSN_MA_2	168	18956.64	8.38	63.24	67.38	-0.687	Nucleus
CSN_MA_20	168	18937.58	6.85	62.91	70.3	-0.654	Chloroplast
CSN_MA_21	219	24849.17	5.49	37.42	83.29	-0.631	Nucleus
CSN_MA_22	220	25120.44	5.25	36.45	85.55	-0.628	Nucleus
CSN_MA_23	227	25629.2	9	51.22	81.23	-0.616	Nucleus
CSN_MA_24	226	25542.17	9.13	49.65	81.59	-0.617	Nucleus
CSN_MA_25	230	25986.48	8.93	50.65	79.74	-0.657	Nucleus
CSN_MA_26	227	25614.11	9.34	50.63	78.24	-0.673	Nucleus
CSN_MA_27	225	25195	9.28	56.28	68.04	-0.546	Chloroplast
CSN_MA_28	225	25249.07	9.08	51.3	68.49	-0.54	Nucleus
CSN_MA_29	82	9242.79	9.42	40.21	84.39	-0.304	Cytoplasm
CSN_MA_3	79	8872.27	9.57	38.01	82.66	-0.347	Nucleus
CSN_MA_30	149	17480.88	9.38	58.36	71.88	-0.983	Nucleus
CSN_MA_31	158	17967.31	6.05	57.14	84.56	-0.53	Chloroplast
CSN_MA_32	178	19802.23	6.05	44.26	81.18	-0.602	Chloroplast
CSN_MA_33	118	13015	9.73	42.35	81.1	-0.107	Chloroplast
CSN_MA_34	118	12961.93	9.51	38.63	84.41	-0.03	Chloroplast
CSN_MA_35	189	21654.97	9.9	55.29	76.93	-0.649	Nucleus
CSN_MA_36	210	24270.88	9.44	54.94	81.76	-0.721	Nucleus
CSN_MA_4	210	24217.64	9.15	53.34	79	-0.745	Nucleus
CSN_MA_5	188	21499.39	8	58.24	80.43	-0.701	Nucleus
CSN_MA_6	111	12649.48	9.81	35.74	79.91	-0.666	Cytoplasm
CSN_MA_7	187	21471.43	9.35	47.07	74.65	-0.816	Cytoplasm
CSN_MA_8	191	21577.45	6.32	46.44	69.01	-0.764	Chloroplast
CSN_MA_9	191	21564.39	5.99	49.55	66.44	-0.747	Chloroplast
CSN_MB_1	261	29063.07	8.66	39.23	70.27	-0.663	Nucleus
CSN_MB_10	261	29153.2	8.7	39.23	70.27	-0.668	Nucleus
CSN_MB_11	271	31531.63	8.89	34.31	72.32	-0.469	Chloroplast
CSN_MB_12	295	33972.62	8.47	27.48	76.34	-0.328	Vacuole
CSN_MB_2	315	36264.75	9.31	23.84	69.62	-0.505	Nucleus
CSN_MB_3	315	36320.8	9.45	22.42	68.98	-0.539	Nucleus
CSN_MB_4	602	68135.48	8.4	40.68	87.76	-0.165	Nucleus
CSN_MB_5	601	68291.63	8.4	41.06	86.61	-0.181	Nucleus
CSN_MB_6	286	32202.47	5.57	42.58	73.25	-0.682	Cytoplasm
CSN_MB_7	283	31891.2	5.37	47.74	71.63	-0.64	Cytoplasm
CSN_MB_8	273	31127.56	4.5	37.99	72.05	-0.592	Nucleus
CSN_MB_9	275	31184.61	4.46	36	71.53	-0.579	Chloroplast
CSN_MC_1	177	20411	9.76	44.24	78.25	-0.605	Nucleus
CSN_MC_10	177	20376.84	9.76	45.09	74.97	-0.676	Nucleus
CSN_MC_2	462	50254.45	7.76	53.72	71.17	-0.652	Cytoplasm
CSN_MC_3	446	48440.38	6.88	52.2	71.12	-0.646	Nucleus

CSN_MC_4	237	27186.28	9.37	56.77	67.47	-0.713	Nucleus
CSN_MC_5	237	27168.25	9.37	53.19	68.69	-0.706	Nucleus
CSN_MC_6	237	27139.12	9.3	56.5	67.89	-0.723	Nucleus
CSN_MC_7	237	27199.28	9.3	57.18	69.96	-0.691	Nucleus
CSN_MC_8	233	27104.43	9.52	59.19	67.77	-0.688	Nucleus
CSN_MC_9	233	27060.41	9.5	58.56	68.63	-0.674	Cytoplasm
CSN_MIKC_1	212	24714.37	8.55	63.39	78.21	-0.704	Chloroplast
CSN_MIKC_10	212	24763.36	8.55	66.67	76.37	-0.73	Nucleus
CSN_MIKC_11	351	40167.15	5.37	58.9	80.23	-0.655	Nucleus
CSN_MIKC_12	355	40810.86	5.52	56.83	79.32	-0.676	Chloroplast
CSN_MIKC_13	218	25027.12	9.92	48.79	86.83	-0.649	Chloroplast
CSN_MIKC_14	168	19700.01	10.19	56.67	84.17	-0.952	Nucleus
CSN_MIKC_2	360	40632.77	6.12	48.17	78	-0.613	Chloroplast
CSN_MIKC_3	360	40519.61	6.12	48.18	76.67	-0.614	Nucleus
CSN_MIKC_4	351	39590.79	6.76	50.91	77.75	-0.61	Nucleus
CSN_MIKC_5	362	40695.2	8.33	47.49	80.5	-0.5	Cytoplasm
CSN_MIKC_6	929	102014.02	5.26	56.79	75.62	-0.573	Chloroplast
CSN_MIKC_7	313	35231.38	5.3	52.8	78.82	-0.636	Nucleus
CSN_MIKC_8	507	57269.47	6.15	42.48	67.53	-0.825	Nucleus
CSN_MIKC_9	500	56605.89	6.22	38.99	69.24	-0.813	Nucleus
CSN_PI_1	348	41241.76	6.95	57.4	79.34	-1.056	Nucleus
CSN_PI_2	348	41200.7	6.73	57.95	79.34	-1.045	Chloroplast
CSN_SEP_1	244	27814.8	8.45	49.84	84.39	-0.596	Nucleus
CSN_SEP_10	244	27786.74	8.45	50.97	83.98	-0.595	Nucleus
CSN_SEP_2	243	27894.81	9.01	41.84	83.09	-0.674	Chloroplast
CSN_SEP_3	243	27954.86	9.01	41.82	82.67	-0.686	Chloroplast
CSN_SEP_4	250	28610.71	8.99	51.9	86.6	-0.575	Nucleus
CSN_SEP_5	250	28587.75	8.99	53.12	88.16	-0.536	Nucleus
CSN_SEP_6	250	28587.75	8.99	53.12	88.16	-0.536	Nucleus
CSN_SEP_7	244	27907.54	7.8	52.4	81.6	-0.716	Chloroplast
CSN_SEP_8	244	27913.54	7.8	52.64	80	-0.717	Nucleus
CSN_SEP_9	244	27913.54	7.8	52.64	80	-0.717	Nucleus
CSN_SOC1_1	212	24945.47	8.82	53.99	76.84	-0.88	Mitochondrion
CSN_SOC1_2	212	24892.42	8.53	56.15	76.84	-0.847	Nucleus
CSN_SOC1_3	220	25188.71	9.24	53.41	75.82	-0.862	Nucleus
CSN_SOC1_4	202	23516.12	9.23	53.06	83.02	-0.772	Nucleus
CSN_SOC1_5	202	23517.1	9.11	53.72	83.02	-0.772	Nucleus
CSN_SVP_1	718	81310.28	6.01	40.06	81.04	-0.51	Nucleus
CSN_SVP_2	730	82701.86	6.25	39.3	79.71	-0.531	Nucleus
CSN_SVP_3	238	27570.06	10.11	57.33	90.55	-0.521	Nucleus
CSN_SVP_4	227	25359.96	8.6	59.8	88.9	-0.527	Nucleus
CSN_SVP_5	217	24674.98	6.18	57.03	87.19	-0.693	Nucleus
CSN_SVP_6	217	24646.96	6.18	56.63	87.19	-0.69	Chloroplast

CSN_TT16_1	211	24384.99	9.39	61.2	86.87	-0.681	Nucleus
CSN_TT16_2	211	24368.98	9.39	62.06	88.72	-0.69	Vacuole
CSN_TT16_3	240	28173.99	7.22	58.12	84.12	-0.813	Nucleus
CSN_TT16_4	240	28173.99	7.22	58.12	84.12	-0.813	Chloroplast
CSN_XALI_1	201	22943.76	8.36	55.97	92.24	-0.296	Nucleus
CSN_XALI_2	201	22901.67	7.68	55.6	91.74	-0.292	Cell membrane

Supplementary Table 6: **Comparison of RNA-seq and qPCR data**

Gene ID	Gene name	log2FC(RNA-seq)	log2FC(QPCR)
evm.TU.Chr3.2090	CSN_SEP_3	2.94	1.8297
evm.TU.Chr8.2342	CSN_AG_6	1.62	2.784
evm.TU.Chr6.170	WUS	-4.04	-2.595
evm.TU.Chr1.1997	CSH_AG_8	3.55	2.7675
evm.TU.Chr8.1346	CSH_AG_4	0.91	1.2518
evm.TU.Chr1.1118	CSH_AP1-FUL_2	-0.27	-1.128
evm.TU.Chr1.4019	CSH_AGL6_4	-1.8	-0.9485
evm.TU.Chr2.1893	CSH_PI_2	-0.26	1.1195
evm.TU.Chr1.1117	CSH_AP1-FUL_1	-1.67	-2.1465
evm.TU.Chr1.1092	/	-2.88	-2.5375
evm.TU.Chr1.3823	/	-1.7	-1.216
evm.TU.Chr1.4125	/	-3.06	-2.0071
evm.TU.Chr1.2908	/	-2.26	-3.0802
evm.TU.Chr2.1619	/	-0.27	-0.2188
evm.TU.Chr5.1540	/	1.32	2.3045

Supplementary Table **7: Hormone profiling data of Newhall**

Hormone	N-T1-1	N-T1-2	N-T1-3	N-T2-1	N-T2-2	N-T2-3	N-T3-1	N-T3-2	N-T3-3
TRA	556.539181	598.767998	573.324639	555.691097	544.065384	543.032086	282.345877	224.28761	215.387421
JA-Val	0.747189407	0.607204421	0.812367075	170.068214	150.453318	168.713539	1.13609888	0.953640825	0.880426853
IAN	0.86720319	0.861795275	1.20689958	88.5622884	95.082957	83.0763428	58.4372498	52.2884115	48.9328667
OPDA	29.403672	23.8139576	32.4596208	298.117905	261.545258	329.820459	54.7207766	41.3892228	38.331526
JA	77.7741681	62.9066112	78.067817	5330.8106	4895.00295	5319.10078	97.3601381	90.94376	87.0231171
ICAld	6.02320831	5.86326303	7.31400482	84.5010954	81.489299	73.9961778	14.1660328	19.6655148	18.6111002
JA-ILE	41.6290116	34.0098357	39.2398174	664.240191	614.865993	673.440958	30.0232686	26.1696214	24.7244677
IAA-Phe-Me	0.195752117	0.213721862	0.220452949	0.584666899	0.741274789	0.974655502	2.37744195	2.26298077	2.23783517
TRP	20438.9151	14351.6528	16982.6695	223941.944	199356.47	236296.52	165070.306	147539.563	171282.285
MEIAA	0.698326442	0.693485094	0.755482544	8.60458574	7.86277734	7.46736069	4.02354384	3.93627304	4.06863663
IAA-Asp	6.40893877	7.81887548	8.45420345	3.10488449	3.01018555	2.90027158	1.34985739	1.26047676	1.32840842
2MeScZR	0.159920752	0.156953458	0.193133026	0.875368452	0.944875319	0.910671897	1.20914732	1.36804888	1.34999014
IP	0.311107009	0.285806632	0.332436296	0.621571898	0.667443059	0.591659123	0.233058947	0.28747496	0.249181782

2-Coumarate	13372.0467	13518.911	11669.9689	12065.4252	10553.3575	11493.7387	2567.05364	2594.1256	2745.04633
GA24	2.69560937	2.93142872	2.804374	2.70496415	2.56717554	2.38790485	6.89198859	6.22218049	6.55274054
SA	102.503199	96.8540864	113.850321	76.3739793	64.6276752	59.1402635	88.8523319	99.85001	96.2946076
Phe	5444.1327	5454.26891	5199.61376	13667.5214	12817.3473	13044.7596	8989.29143	10835.7873	11027.1836
GA3	1.04324424	1.11199807	0.919861055	3.41289086	3.71750442	3.51672199	5.71629804	8.62232071	8.71374704
ABA	161.293562	161.597546	146.435343	302.643896	289.262223	285.928385	732.698659	838.476562	893.560233
DZ	0.752187931	0.673841513	0.736321228	1.20300239	1.01603917	1.0541214	0	0	0
IPR	0	0	0	9.52412368	10.2634007	13.2458761	1.57764462	1.35798528	1.20726784
tZOG	5.59499902	5.46950416	6.91201846	9.28589922	8.75456509	9.87112754	77.3588871	61.6018129	64.1445682
iPRMP	8.4381522	10.4662594	7.39985453	19.396435	18.0124926	12.1069453	0	0	0
MEJA	0	0	0	513.657638	539.036423	528.070308	13.5646767	10.9739333	13.2049487
IAA-Leu-Me	0	0	0	0.690123979	0.750628313	0.617637296	0	0	0
GA9	0	0	0	6.08601374	5.23785097	4.28017502	6.18464772	5.82371795	6.19390773
ICA	0	0	0	31.1212906	27.9007952	27.3823174	8.87640112	11.0635517	13.0186071
OPC-4	0	0	0	0	0	0	57.5823159	45.8588742	47.6227326
DHZROG	0	0	0	0.210012946	0.200316611	0.213827701	0.182460969	0.169032953	0.179071372
IAA	14.6683648	17.5617522	16.8987259	23.3229934	23.8357304	21.3841028	0	0	0

cZROG	0	0	0	3.47948616	3.6419841	3.45511467	2.07689151	2.01226713	1.91410686
cZR	0.257875566	0.267825999	0.32578752	0	0	0	0.828119996	0.724251302	0.765109917
cZRMP	2.82090471	2.71007402	2.26758126	4.70812587	4.40221873	3.75744317	0	0	0
tZR	0	0	0	44.4007668	51.8807677	53.5148863	0	0	0
JA-Phe	0	0	0	34.2070803	31.1682702	32.8867431	1.16226481	0.995385116	0.937295446
tZRMP	0	0	0	106.075732	101.605144	68.2993864	0	0	0
tZ	0	0	0	2.65240988	2.92749853	2.75862503	0	0	0
GA29	0	0	0	0	0	0	0	0	0
GA19	0	0	0	0	0	0	0	0	0
IAA-Val-Me	0	0	0	8.94463254	6.00763303	8.67038825	0	0	0
H2JA	0	0	0	5.46186019	5.68167092	5.4900171	0	0	0
MeSAG	0	0	0	0	0	0	0	0	0
GA20	0	0	0	0	0	0	0	0	0

Supplementary Table **8: Hormone profiling data of Hamlin**

Hormone	H-T1-1	H-T1-2	H-T1-3	H-T2-1	H-T2-2	H-T2-3	H-T3-1	H-T3-2	H-T3-3
TRA	140.518269	150.659528	161.436224	312.105211	278.646993	274.354694	220.878074	231.013988	255.360697
JA-Val	0.412303033	0.379233387	0.506579082	100.015298	109.321472	106.259219	0.998099385	0.864363385	0.997462687
IAN	1.25720652	1.24030725	1.27740051	92.6320075	76.9695282	81.6371836	27.8048156	28.3748517	24.6234826
OPDA	115.67855	127.497748	130.738776	654.221773	878.148411	787.838848	47.1498463	71.4914492	54.1308458
JA	56.7153339	57.4186849	62.9362245	8577.82274	8848.29925	8806.78194	144.039703	114.047548	143.058706
ICAld	4.98116506	4.90404824	5.29408163	48.8817608	42.8991193	50.0747459	24.9532787	17.6992141	32.0308458
JA-ILE	22.9538852	24.911229	25.1590561	636.527833	704.322737	669.399043	31.6178279	27.6670621	34.9738806
IAA-Phe-Me	0.911421607	1.15044536	1.04333163	1.06192262	1.17007998	1.10039366	2.47345799	2.42939897	2.2171791
TRP	32229.9094	23082.8913	30093.6224	186912.011	183703.432	151934.921	186857.326	207722.42	264883.085
MEIAA	0.890570219	0.835523419	0.804964286	7.99987663	8.61472464	8.20375723	4.96949283	6.03519178	5.66649254
IAA-Asp	3.57393638	3.60283227	2.57540816	4.79424102	4.69272626	4.6960584	1.9732915	1.5936462	1.92624378
2MeScZR	0.245654422	0.319895917	0.240793367	1.41295894	1.565246	1.46916235	1.24957736	1.3617413	1.4715995
IP	0.380771125	0.348193555	0.359869898	0.364436932	0.289835999	0.319157365	0.309316086	0.347674476	0.211186567

2-Coumarate	11147.8728	11170.5364	11523.2398	11454.2045	10137.5785	11440.8013	4028.02254	4161.64986	3782.51244
GA24	7.37423675	6.93409728	6.58627551	4.0403178	3.82180097	4.2110574	8.24951332	9.24906089	9.20393035
SA	132.105328	137.027872	141.072704	63.3063561	60.0179692	51.5644309	142.755891	159.815144	143.803483
Phe	5774.6947	5681.3701	6091.42857	14531.3364	14426.0731	14353.9715	7579.20082	7012.38138	8382.63682
GA3	0.992581741	1.21403373	0.985369898	12.7702823	13.7371938	11.7161152	10.2751793	7.70054864	11.0325622
ABA	184.848828	189.302442	182.824745	384.588433	374.415367	349.867949	611.196209	676.312278	648.350746
DZ	0.954220012	1.049372	1.10361735	0.813158804	0.808683438	0.84436167	0.680796619	0.74477066	0.61621393
IPR	8.78412448	8.86226481	9.20836735	4.13173608	5.53783661	4.74843034	1.59330686	2.24584322	1.60753483
tZOG	5.08572976	6.0638761	6.45260204	8.12968812	7.67852804	8.9431184	0	0	0
iPRMP	15.1660429	14.2556045	15.7307398	9.60817213	8.40015691	8.8909707	1.80179047	2.57579577	2.56751244
MEJA	32.7297124	32.6090873	29.9375	761.949763	688.464264	759.248555	9.81401127	8.75696916	11.5849751
IAA-Leu-Me	0.288100748	0.262580064	0.270477041	0.409906731	0.439848654	0.398318218	0.658913934	0.62577847	0.709912935
GA9	0	0	0	4.76515002	3.95140717	4.70071756	5.4748207	4.34284796	5.23271144
ICA	0	0	0	20.343787	26.5716744	24.7315378	17.1244621	18.1452155	19.2404478
OPC-4	125.189334	137.511009	138.56352	40.3891137	46.7622494	45.8209587	50.3865266	59.2331455	63.2741294
DHZROG	0	0	0	0.247332708	0.265777485	0.276492426	0.311531762	0.288132661	0.267335821
IAA	11.230845	10.605034	11.0201276	19.7449664	24.7378265	23.0854096	0	0	0

cZROG	0	0	0	2.26536222	2.39566208	2.21975782	4.2783043	4.97847469	4.48477612
cZR	0	0	0	3.84775957	4.05656509	4.11697728	1.06744621	1.15073893	1.12337811
cZRMP	0	0	0	3.78397651	3.06152561	2.60040861	0	0	0
tZR	4.91094643	4.37344876	5.04135204	20.8294266	21.7745495	20.3253687	0	0	0
JA-Phe	0	0	0	76.7580438	95.7139603	83.6954355	0	0	0
tZRMP	0	0	0	62.5180122	45.8913748	48.4839047	0	0	0
tZ	0	0	0	1.91642568	1.80829621	1.66308302	0	0	0
GA29	0	0	0	49.8228385	49.0812918	44.602352	38.6134734	42.7607256	41.1674129
GA19	4.11515166	4.39434047	4.90645408	4.73201244	3.69672505	3.84348216	0	0	0
IAA-Val-Me	0	0	0	9.58409001	10.6548137	10.1074347	0	0	0
H2JA	0	0	0	7.88489439	8.33802389	8.46148096	0	0	0
MeSAG	0	0	0	128.851411	139.687437	133.345874	0	0	0
GA20	18.2758765	19.7038381	23.157449	0	0	0	0	0	0
